# A Quantitative High-Resolution Genetic Profile Rapidly Identifies Sequence Determinants of Hepatitis C Viral Fitness and Drug Sensitivity

**DOI:** 10.1371/journal.ppat.1004064

**Published:** 2014-04-10

**Authors:** Hangfei Qi, C. Anders Olson, Nicholas C. Wu, Ruian Ke, Claude Loverdo, Virginia Chu, Shawna Truong, Roland Remenyi, Zugen Chen, Yushen Du, Sheng-Yao Su, Laith Q. Al-Mawsawi, Ting-Ting Wu, Shu-Hua Chen, Chung-Yen Lin, Weidong Zhong, James O. Lloyd-Smith, Ren Sun

**Affiliations:** 1 Department of Molecular and Medical Pharmacology, University of California Los Angeles, Los Angeles, California, United States of America; 2 The Molecular Biology Institute, University of California Los Angeles, Los Angeles, California, United States of America; 3 Department of Ecology and Evolutionary Biology, University of California Los Angeles, Los Angeles, California, United States of America; 4 Department of Molecular, Cell and Developmental Biology, University of California Los Angeles, Los Angeles, California, United States of America; 5 Department of Human Genetics, University of California Los Angeles, Los Angeles, California, United States of America; 6 Institute of Information Science, Academia Sinica, Taipei, Taiwan; 7 Institute of Biomedical Informatics, National Yang-Ming University, Taipei, Taiwan; 8 Department of Infectious Diseases, Novartis Institutes for BioMedical Research, Emeryville, California, United States of America; 9 Fogarty International Center, National Institutes of Health, Bethesda, Maryland, United States of America; 10 School of Medicine, Zhejiang University, Hangzhou, Zhejiang, China; University of Texas at Austin, United States of America

## Abstract

Widely used chemical genetic screens have greatly facilitated the identification of many antiviral agents. However, the regions of interaction and inhibitory mechanisms of many therapeutic candidates have yet to be elucidated. Previous chemical screens identified Daclatasvir (BMS-790052) as a potent nonstructural protein 5A (NS5A) inhibitor for Hepatitis C virus (HCV) infection with an unclear inhibitory mechanism. Here we have developed a quantitative high-resolution genetic (qHRG) approach to systematically map the drug-protein interactions between Daclatasvir and NS5A and profile genetic barriers to Daclatasvir resistance. We implemented saturation mutagenesis in combination with next-generation sequencing technology to systematically quantify the effect of every possible amino acid substitution in the drug-targeted region (domain IA of NS5A) on replication fitness and sensitivity to Daclatasvir. This enabled determination of the residues governing drug-protein interactions. The relative fitness and drug sensitivity profiles also provide a comprehensive reference of the genetic barriers for all possible single amino acid changes during viral evolution, which we utilized to predict clinical outcomes using mathematical models. We envision that this high-resolution profiling methodology will be useful for next-generation drug development to select drugs with higher fitness costs to resistance, and also for informing the rational use of drugs based on viral variant spectra from patients.

## Author Summary

The emergence of drug resistance during antiviral treatment limits treatment options and poses challenges to pharmaceutical development. Meanwhile, the search for novel antiviral compounds with chemical genetic screens has led to the identification of antiviral agents with undefined drug mechanisms. Daclatasvir, an effective NS5A inhibitor, is one such example. In traditional methods to identify critical residues governing drug-protein interactions, wild type virus is passaged under drug treatment pressure, enabling the identification of resistant mutations evolved after multiple viral passages. However, this method only characterizes a fraction of the positively selected variants. Here we have simultaneously quantified the relative change in replication fitness as well as the relative sensitivity to Daclatasvir for all possible single amino acid mutations in the NS5A domain IA, thereby identifying the entire panel of positions that interact with the drug. Using mathematical models, we predicted which mutations pose the greatest risk of causing emergence of resistance under different scenarios of treatment compliance. The mutant fitness and drug-sensitivity profiles obtained can also inform the patient-specific use of Daclatasvir and may facilitate the development of second-generation drugs with a higher genetic barrier to resistance.

## Introduction

The rise of drug resistance to antimicrobial agents, frequently a consequence of acquiring mutations de novo that confer resistance, causes failure of current infectious disease treatments and results in continued economic burden [1]. Resistance development is an evolutionary process, often depending on the combined effects of fitness cost and resistance gain of the associated mutations [2], [3]. It is of paramount importance to systematically explore the evolutionary dynamics of infectious pathogens to assess the likelihood of resistance breakthrough during drug development. In this study, we utilize Hepatitis C virus (HCV) and a potent antiviral compound (Daclatasvir) as a working model to illustrate the application of a quantitative high-resolution genetic (qHRG) platform to interrogate the Daclatasvir-resistance profile of the virus.

Persistent infection with HCV is a major cause of human liver damage in over 3% of the world's population, and consequently, these patients are at risk of developing advanced liver diseases and liver cancer [4]. The long-standard treatment for HCV infection is a combination of ribavirin and PEGylated interferon (PEG-IFN), which activates the immune system and thus causes severe side effects [5]. Ever since the discovery of HCV almost 25 years ago, enormous effort has been devoted to understanding the replication life cycle of the virus and developing effective direct-acting antiviral (DAA) drugs with the goal of reducing its global health impact. In 2011, the first two protease inhibitors were approved and used in combination with standard treatment [6], [7]. Although the viral response rate in patients has markedly improved with the addition of the protease inhibitors, the efficacy of this new therapeutic regimen is observed to be highly dependent on HCV genotype. Moreover, the emergence of resistant mutations further hinders its application [8], [9] and creates a demand for more effective treatment options.

The establishment of the HCV replicon cell [10], [11] and infectious [12]–[14] systems have paved the way for high-throughput screening of small-molecule inhibitors, and thereby aided the identification of many new classes of antiviral compounds [15], [16]. However, the ability to systematically define mechanisms of action and determine the genetic barriers of promising compounds poses unmet challenges [17], [18]. Previously, a chemical screen has identified potent antiviral compounds that target the HCV protein NS5A [19], [20], which is a non-enzymatic protein but is indispensable for viral genome replication, viral assembly, and innate immune evasion [21]. The mechanism of action and binding site of many NS5A inhibitors, however, remain unknown. The NS5A inhibitor Daclatasvir was identified as a potent antiviral agent that blocks viral replication at both the genome replication and viral assembly stages [22]. It possesses a potent antiviral activity in cell culture, with a half maximal effective concentration (EC_50_) in the pico molar (pM) range and a cytotoxic concentration in the micro molar (uM) range, yielding a large potential therapeutic window [20]. Daclatasvir has also been reported to alter the localization of NS5A [23], but the mechanism of drug-protein interactions is under investigation and not yet fully understood [22]. Moreover, considering the fast replication rate and error-prone RNA polymerase of HCV, drug escape mutants are expected. Therefore, a systematic investigation of the evolutionary fitness and Daclatasvir sensitivity of all possible variants is imperative to better understand the mechanism of drug action and to design second-generation compounds with higher genetic barriers for resistance. Previous studies passaging wild type (WT) HCV clones in the presence of Daclatasvir identified resistant mutations within the NS5A domain IA (DIA), suggesting an interaction between the drug and this region [19], [20], [24]–[26]; however such studies are limited by the breadth of genetic variability and can only identify positively selected mutations.

Here we have implemented a qHRG platform to simultaneously quantify the effects of all possible single mutations in NS5A DIA on relative replication fitness and sensitivity to Daclatasvir. Our dataset includes all NS5A DIA mutations that both increase and decrease drug sensitivity at any magnitude and therefore provides an informative framework for identifying residues and key chemical contacts that may be involved in protein recognition of the drug. Quantitative analysis of the altered drug sensitivity for each mutant enabled determination of residues mediating drug-protein interactions. Complete analysis of drug sensitivity for all possible single amino acid variants also greatly clarifies the differences in Daclatasvir sensitivity among different HCV genotypes. Moreover, the relative fitness and drug sensitivity profiles generated by qHRG were further utilized to predict genotype specific clinical outcomes using mathematical models. We anticipate that the qHRG approach will be generally applicable to studying other virus-host interactions or drug-protein interactions to understand the underlying mechanisms of drug action.

## Results

### Fitness landscape of NS5A domain IA

**Figure 1:**
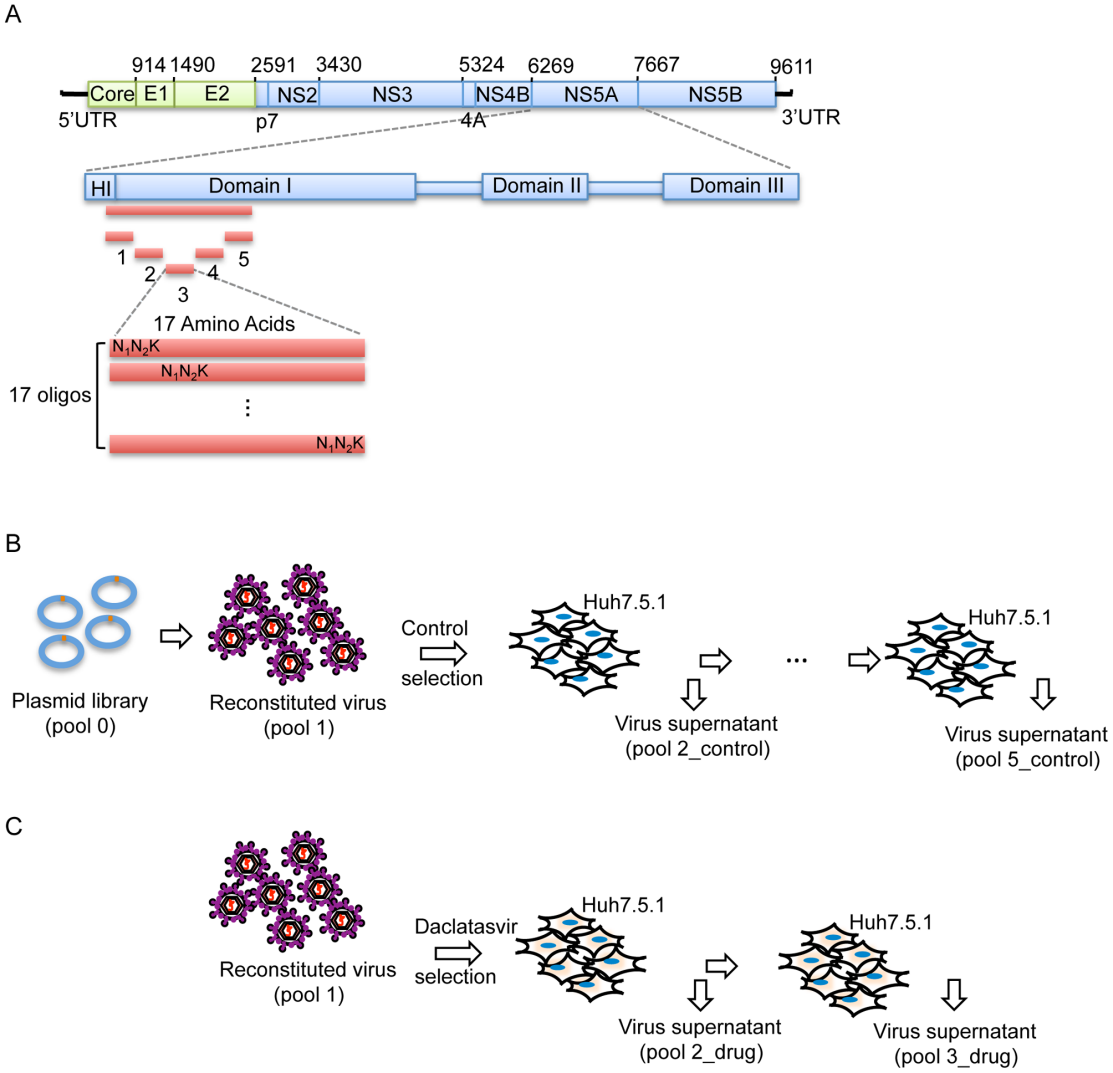
Saturation mutant library construction and selections. (A) Schematic picture showing the construction of the saturation mutant library in a sub-domain of NS5A of HCV. The area to be mutated was divided into 5 small regions, and each of them was composed of 17 or 18 amino acids. Each residue was replaced with one random codon (N_1_N_2_K: N_1_ and N_2_ codes for A/T/G/C and K codes for T/G) and incorporated into the WT background of HCV. The resultant viral library was then selected in the absence of Daclatasvir for 4 rounds (B) or in the presence of drug (20 pM of Daclatasvir) for 2 rounds (C).

To systematically map the fitness landscape and assess the drug-resistance profile of NS5A DIA, saturation mutagenesis techniques were used to introduce randomization at each codon position (amino acids 18–103) thereby covering all possible amino acid substitutions. To do this, we substituted each codon individually using synthetic template oligonucleotides that contained 3 continuous random nucleotides ‘N_1_N_2_K’ at the codon of interest, where N_1_ and N_2_ represent random incorporation of A/T/G/C, and K represents random incorporation of T/G. The randomized codons therefore include 32 nucleotide combinations, allowing representation of all possible amino acids at each position (Fig. 1A). The input DNA library (pool 0, Fig. 1B) was isolated from more than 21,000 bacteria colonies to ensure coverage of all possible mutations in the pool 0 (Table S1A). The viral library (pool 1) was reconstituted and subsequently subject to selection in a human hepatocyte cell line (Huh-7.5.1) for 4 rounds (pool 2_control through pool 5_control) of infection at a low multiplicity of infection (MOI) (Fig. 1B, Table S1B). Pools labeled “control” indicate replication without the presence of drug. The fitness of each mutant, reflected by the change in frequency of each mutation, was determined by Illumina paired-end sequencing, allowing for the identification and quantification of all mutants with high confidence [27].

**Figure 2:**
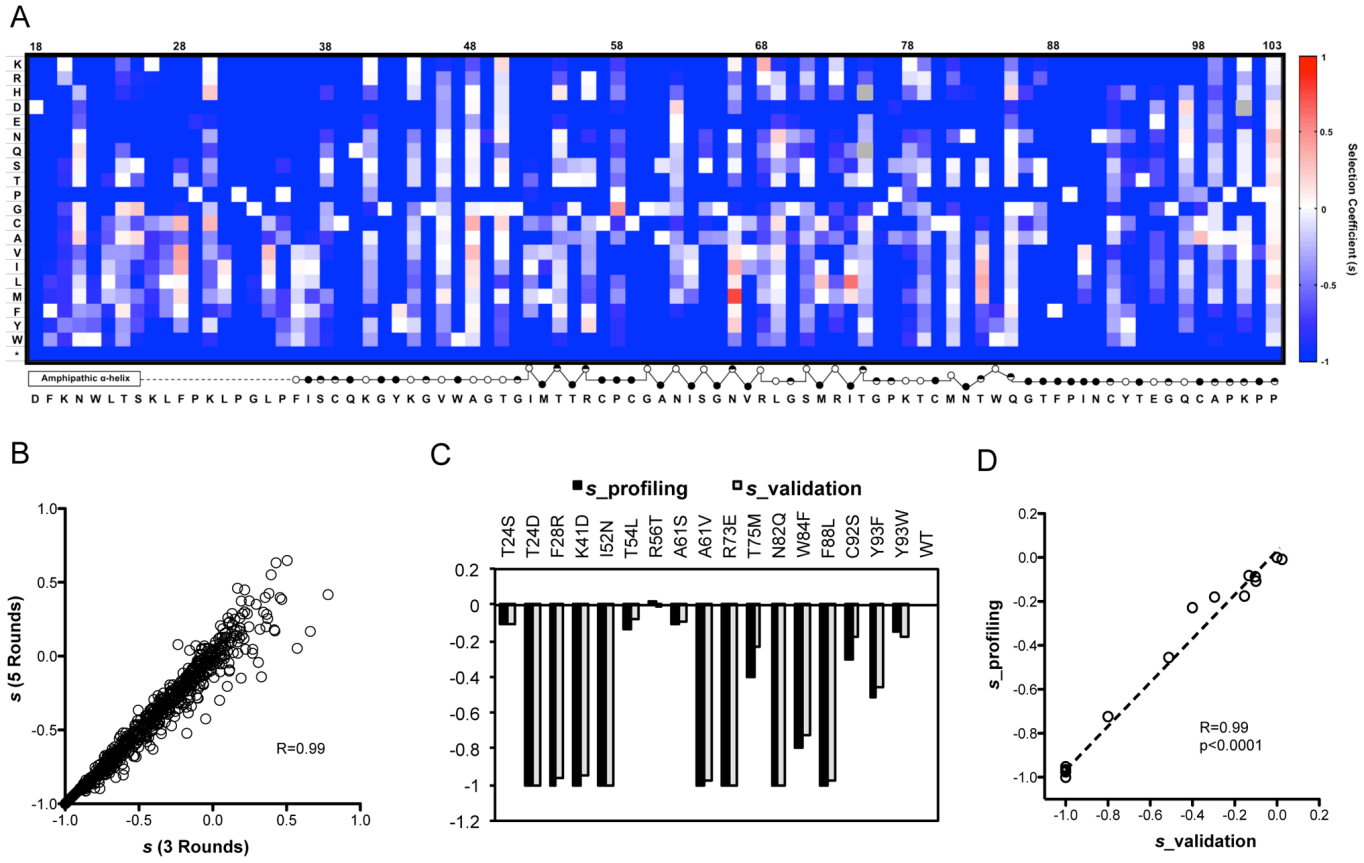
The fitness landscape of amino acids 18–103 in NS5A in virus replication. (A) A heat map showing the profile of relative fitness represented as selection coefficient (*s*) for each variant during viral replication *in vitro*. Color indicates the fitness of each mutant calculated as *‘s’* relative to WT (Materials and method). Red represents positive *‘s’* (*i.e.* increased fitness) and blue represents negative *‘s’*. *s* = 0 means the same fitness as the WT virus. The secondary structure of the mutated region is annotated below the figure (open circles: solvent exposed residues; filled circles: buried residues; half-filled circles: partially buried residue). (B) Selection coefficient (*s*) of individual point mutations (relative to WT) calculated from 5 rounds of selection (*s* (5 Rounds)) shows a strong correlation with *s* values calculated from 3 rounds of selection (*s* (3 Rounds)). (C) Validation of the fitness measurements from profiling using individually constructed mutant viruses. (D) The selection coefficients of individual mutants in the validation experiment correlate strongly (R = 0.99) and significantly (p<0.0001) with *s* values derived from qHRG profiling.

From the 23.9 million sequence reads that passed quality filtration, each mutant virus was sequenced approximately 1200 times on average, which enabled precise quantification of mutant frequencies. The WT fraction served as an internal benchmark to determine the relative fitness of each mutant. The relative fitness score (*W*) of each mutant was determined by regression analysis of the mutant frequency relative to WT through 5 rounds of selection [28]. The selection coefficient (*s*) of each variant was also calculated (Methods). Interestingly, as shown in figure 2B, the selection coefficients calculated from 5 rounds of selection (pool 1 and pool 2–5_control) strongly correlate with those calculated from 3 rounds of selection (pool 1, pool 2_control and pool 3_control), suggesting that 3 rounds of passaging is sufficient to determine the phenotype of each variant. The selection coefficients (*s*) representing the difference in fitness between all variants and WT are displayed in a heat map representation (Fig. 2A).

In agreement with the critical functions of NS5A required for viral replication, stop codons are not tolerated at any position of the region (Fig. 2A), which demonstrates the effectiveness of our selection assay and its reliability in measuring changes in frequency. To verify the accuracy of our fitness profiling method, 16 mutant viruses that span the range of all phenotypes and span a range of functional and structural motifs were constructed on a monocistronic Renilla luciferase HCV reporter virus background (FNX24_RLuc). A reporter virus defective in RNA polymerase activity (NS5B_GNN contains a double mutation within the RNA-dependent RNA polymerase motif of NS5B that converts GDD into GNN) served as a negative control [29] and WT as a positive control. The individually determined selection coefficients show strong correlation at high confidence with the profiling data (Fig. 2C, D), demonstrating the accuracy of fitness measurements from the qHRG profile throughout a large dynamic range.

### High-resolution profiling of NS5A domain IA reveals residues critical for virus replication

**Figure 3:**
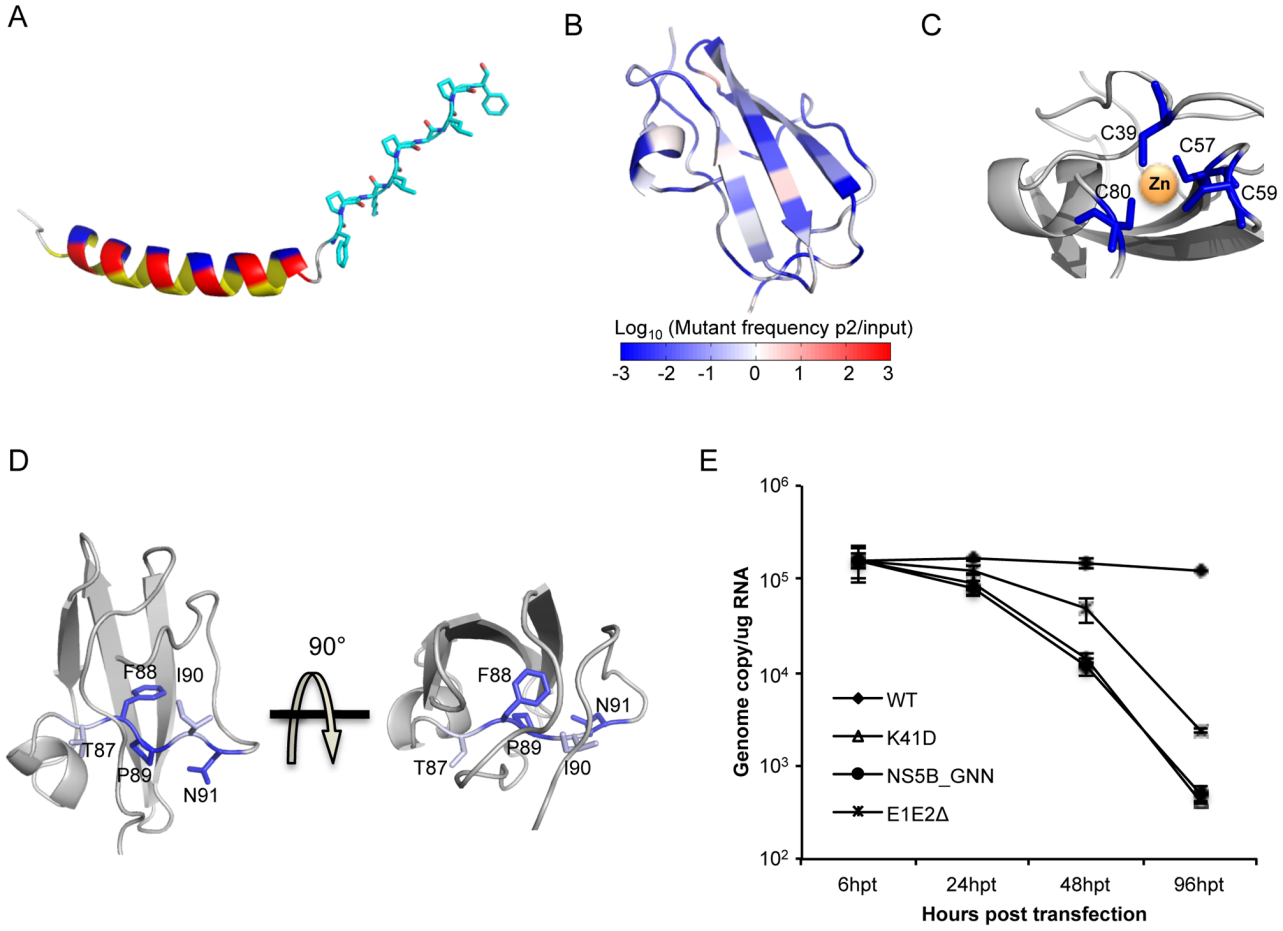
High-resolution genetics revealed functional residues essential for virus replication. (A) Color-coded structure illustrating the NMR model of the helix and the linker region (shown in sticks) modeled as the poly-proline II helix. The three faces of the helix are highlighted in yellow (hydrophobic), red (acidic) and blue (non-acidic). (B) Heat map of the protein structure representing the essentialness of each position during virus replication. The fold change of mutations (log_10_) in pool 5_control at each position was projected on a ribbon model of the protein structure using PyMOL. Fold change values were represented by a blue-white-red color map. The color spectrum standard bar is shared between B, C and D. (C) Zinc-binding cysteines are all essential and do not tolerate mutations. The zinc-associated cysteines are colored based on the color scale in (A), and the rest of the region was colored in grey. (D) The conserved stretch of residue 87–91 (highlighted with sticks) in the structure of NS5A DIA. (E) Genome replication measured in transfected Huh7.5.1 cells at 6 h, 24 h, 48 h and 96 h post transfection for WT, K41D, polymerase-inactive mutant (NS5B_GNN) and envelope-protein-deleted mutant (E1E2Δ).

The fitness effects enable fine mapping of sequence-function requirements at each position. For example, the N-terminus forms an amphipathic membrane-binding α-helix and we observe sequence requirements in agreement with the three distinct faces (hydrophobic, acidic, and polar/non-acidic) as determined by NMR structural analyses (Fig. 2C, D, 3A) [30]. Strict sequence requirements at positions within this helix may indicate that this region contributes to the localization of NS5A [31]–[34]. Continuing this trend, the unresolved proline-rich linker region displays a requirement for the sequence KXΦPXΨPGΨP. We illustrated the NMR model of the helix [30] in combination with the linker region modeled as the ubiquitous poly-proline type II helix recognition motif (Fig. 3A) [35], [36].

Our profiling method also identifies strict structural requirements for the unique NS5A DIA fold. In line with an essential structural property [37], four zinc-associated cysteine residues [37] do not tolerate any substitutions (Fig. 2A, 3C). Furthermore, residues 87–91 encompass a conserved segment that passes through the core of the protein (Fig. 3D) which includes a buried polar residue, N91, that does not tolerate any substitutions. F88 and P89 are also absolutely required, while T87 and I90 only tolerate highly similar residues (Fig. 2A). This analysis may suggest potential targets for peptide-based vaccine designs. We also validated that N82 is absolutely essential as even glutamine at this position is lethal (Fig. 2A, C). N82 is buried and participates in hydrogen bonds with both T87 and the Q40 main chain (Fig. 3D, Fig. S1C) highlighting the sensitivity at this position to side chain geometry.

The complete mutagenic analysis of each residue in NS5A DIA also provides in-depth insight into potential molecular recognition surfaces. Although NS5A was previously reported as an RNA-binding protein, the exact binding sites remain to be determined [38]–[40]. Our fitness landscape profile shows that position K41 (R41 in genotype 1b) and K44 exhibit a consistent requirement for WT-like fitness: although these positions tolerate a diverse set of substitutions, acidic residues are lethal at both positions. Substitution with an acidic residue (K41D) results in a defect for genome replication (Fig. 3E). These positions were located in a basic groove large enough to accommodate RNA in one of the dimerized structures [37] (Fig. S2A), providing evidence that these two positions may be important for the RNA binding function of NS5A.

### Drug sensitivity profile reveals novel resistance determinant positions

**Figure 4:**
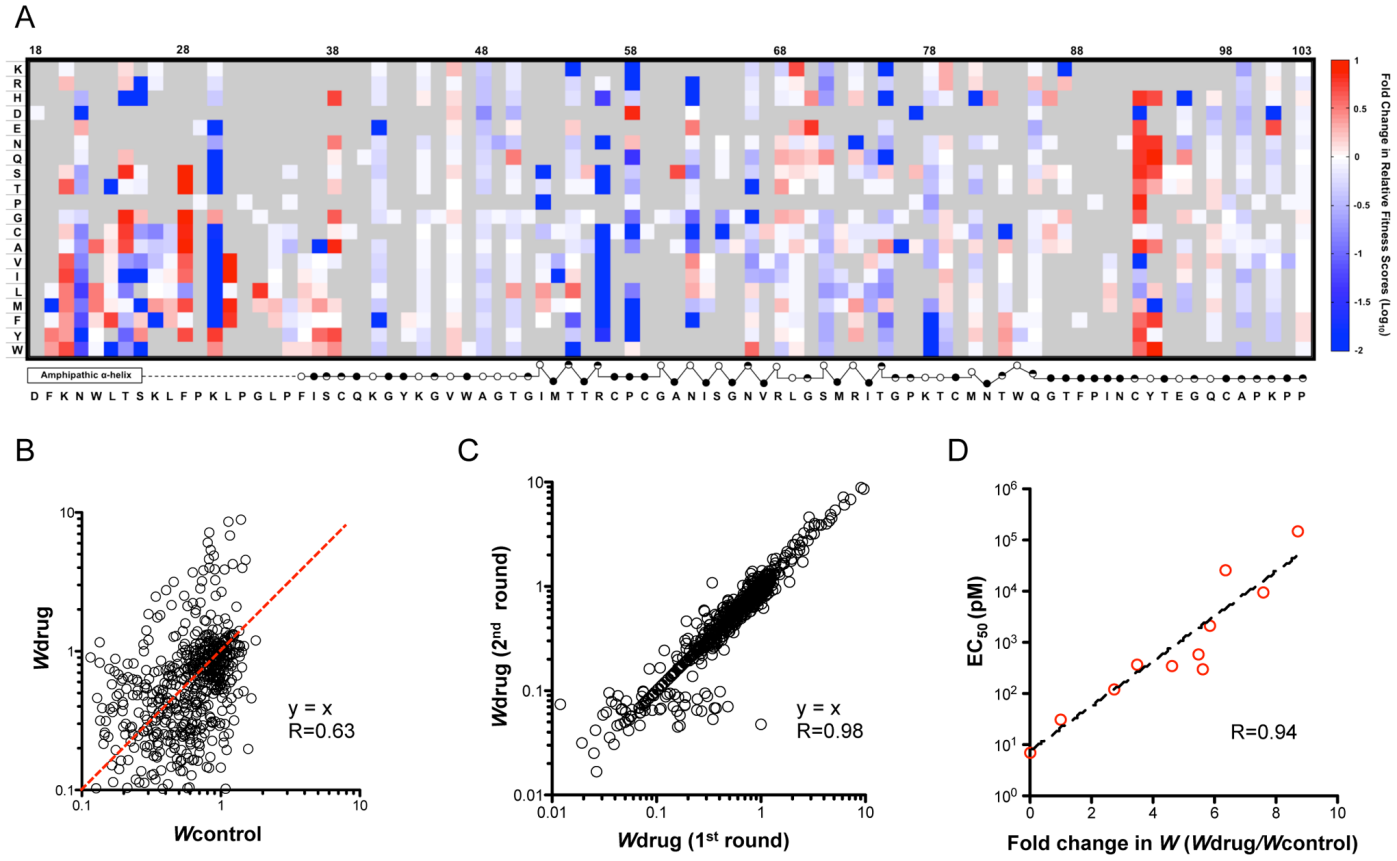
Daclatasvir sensitivity profile and validation. (A) A heat map shows the fitness landscape shift under Daclatasvir treatment (20 pM), which is calculated as the ratio of relative fitness scores (*W*drug/*W*control). The mutants that are lethal without drug are shown in grey. The secondary structure of DIA is annotated below the figure (open circles: solvent exposed residues; filled circles: buried residues; half-filled circles: partially buried residues). (B) Relative fitness scores for individual point mutations in the presence of Daclatasvir (*W*drug) were plotted against the relative fitness scores in the absence of Daclatasvir (*W*control). The red line indicates the same drug sensitivity as WT virus. (C) Strong correlation of *W*drug between the first round and second round of drug selection. *W*drug of each variant in the second round of Daclatasvir selection was plotted against *W*drug in the first round (R = 0.98). (D) Validation of the drug screen results. 10 mutant viruses with a variety of drug sensitivities were reconstructed and their EC_50_ is correlated with the fold of fitness change caused by drug treatment measured in the profiling (R = 0.94).

To quantify changes in drug sensitivity for all non-lethal mutants and to interrogate the drug-protein interaction surface, we passaged the mutant virus libraries under Daclatasvir treatment (20 pM) for 2 rounds (Fig. 1C). The relative fitness score of each mutant in relation to WT under drug treatment (*W*drug) was determined and the selection coefficients are displayed in a heat map (Fig. S3). Next, we calculated the fold change in relative fitness score (*W*drug/*W*control) and the resulting drug sensitivity profile is presented as a heat map in figure 4A. The data show that mutations at positions 28, 31, 38, 92, and 93 are noticeably enriched upon drug selection, suggesting that mutations at these positions confer resistance to the drug. In contrast, the fitness values of variants at positions 21, 56, and 58 are significantly diminished due to increased drug sensitivity. The changes in variant fitness at positions 24, 30, 62, and 75 are highly dependent on the property of the substituted amino acids. We validated the drug sensitivity profile by constructing 10 variants for determination of the Daclatasvir EC_50_ (Fig. S4A, B) and demonstrated an excellent correlation to their drug sensitivity (Fig. 4D) in the profile. The data are also consistent with critical drug-interacting residues identified in previous adaptation experiments [19], [20], [24]–[26]. In addition to corroborating previous results, our profiling method also uncovered new resistance determinants at position 24 and 56 where no resistant mutations were previously identified by virus adaptation studies. Thus, this approach enables identification of all residues participating in the drug interaction network. As a result of our saturation mutagenesis approach, we also identified highly resistant substitutions that were not previously identified at known resistance-conferring positions, including F28C, K30Y, L31I and Y93W. These include substitutions that may require a two-step mutational path in the genetic backgrounds utilized for previous adaptation (e.g. L31I requires two nucleotide changes and has not been observed in genotype 1a, 1b or 2a (JFH1), while M31I only requires one nucleotide change and its breakthrough has been detected in genotype 4a (ED3) [26]). In this study, although we analyzed the *W*drug of each variant from two rounds of selection (pool 2_drug and pool 3_drug in Fig. 1C) to determine the drug sensitivity, the strong correlation of *W*drug between round one and two (Fig. 3C) suggests that a single round of selection is sufficiently informative.

### The drug-sensitivity determinant positions explain genotype-dependent sensitivity of HCV to Daclatasvir

**Figure 5:**
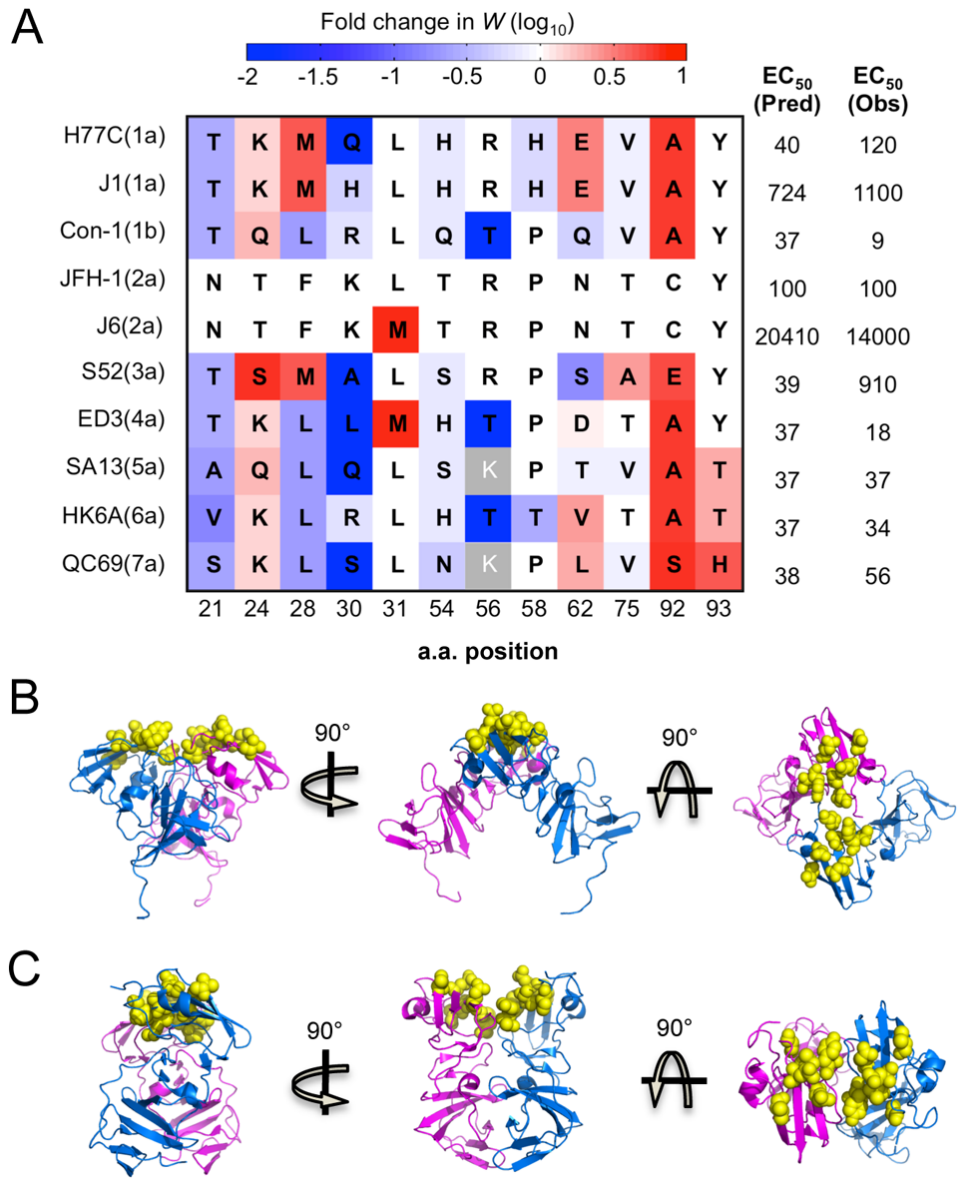
Critical residues mediating drug-protein interactions. (A) 12 residues with altered drug sensitivity are aligned among 10 HCV strains representing 7 genotypes. The drug sensitivity on a genotype 2a background measured in our study, shown as fold change in relative fitness scores, is colored as (4A). For each strain, we predicted the EC_50_ by summing the drug sensitivity effect of residues at all 12 positions, and converting this into EC_50_ (Pred) according to the correlation in Fig. S4C. These predicted values compare well with observed EC_50_ (Obs) values previously determined by experiments [41]. (B) and (C) The drug-associated residues (resolved in structures) clustered together on the surface of the protein. Cartoon diagrams of three rotations of the domain I dimer with residues 54, 56, 58, 62, 75, 92 and 93 highlighted in yellow spheres on PDB structure 1ZH1 [37] (B) and 3FQM [48] (C). Position 21, 24, 28, 30 and 30 are in the helix/linker region, which is not annotated here.

The completeness of our drug sensitivity profile also clarifies the observed differences in Daclatasvir sensitivity among different genotypes recently reported by Scheel *et al.* [41] (Fig. 5A). For instance, the difference between the two 1a strains is likely due to an increased sensitivity of viruses with Q at position 30 compared to H (Fig. 5A). Additionally, our profiling data provides insight into why certain strains that naturally carry residues previously demonstrated to confer resistance in other genetic strains remain highly sensitive to the drug. M31 is a relatively resistant residue according to the drug sensitivity profile and also has been demonstrated to confer significant resistance when acquired in genotype 1a or 1b [24], [42]. However, M31 is the WT residue in the sensitive strain 4a (ED3), suggesting that there are other critical positions involved. Our data indicate that the identity of residue 30 and 56 may contribute to this property as L30 and T56 are much more sensitive than R/K30 and R56. H93 is another high resistance-conferring mutation that is naturally carried in a sensitive genotype 7a strain. This can be explained by the extreme sensitivity of the serine residue at position 30 (Fig. 5A). Likewise, our drug profile shows that T93, found in genotype 5 and 6, is much more resistant than Y93 in genotype 1–4, but this may be largely negated by Q30 in 5a or T56 in 6a. Interestingly, the additive total effect of drug sensitivity from these residues approximates the experimentally determined EC_50_ for 9 out of 10 strains [41] (Fig. 5A). The outlier, genotype 3a (S52), carries residues A30 and E92 which are two positions that have previously been shown to be genetically linked [24], indicating that some of the interactions are epistatic.

All of these analyses demonstrate that the qHRG approach developed in our study can quantitatively determine the drug sensitivity of all point mutations, including the equally informative negatively selected mutations that would not be identified in adaptation selection studies. The results presented here systematically map the entire panel of drug-sensitivity determinants and predict how substitutions will impact HCV replication upon Daclatasvir treatment.

### Clinical outcome prediction using the profiling results

**Figure 6:**
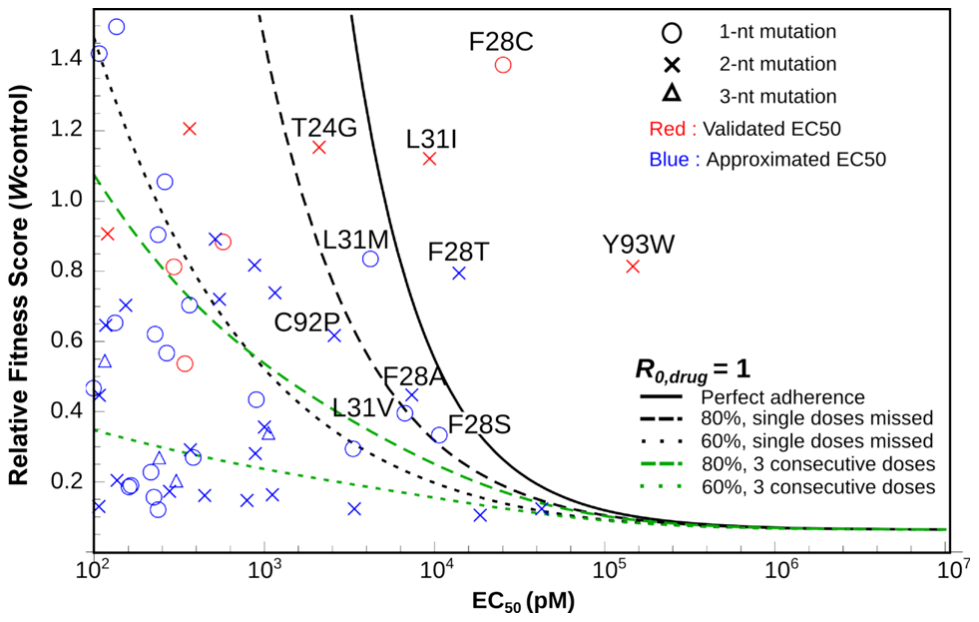
In silico analysis of resistance development and treatment failure for Daclatasvir monotherapy at the recommended clinical dose of 60 Each symbol denotes the measured relative fitness score and measured (red) or predicted (blue) EC_50_ value for a given viral mutant. The lines show where the viral reproductive number, R_0,drug_ equals 1, for a given level of treatment adherence; the colors of the lines denote scenarios where doses are missed randomly as a single dose (black) or in blocks of three consecutive doses (green). Viral mutants above each line are predicted to be resistant to that treatment regimen using the best-estimate parameter values. Labeled mutants are those for which calculations accounting for stochasticity and parameter uncertainties predict >1% probability of therapeutic failure even for perfect adherence (see Fig. S5).

Measurement of drug sensitivity and fitness of all possible point mutations maps the evolutionary space of the virus upon drug treatment and also provides comprehensive information to predict the clinical outcome of single mutations resistant to Daclatasvir treatment for genotype 2a. We combined previous mathematical models of viral evolution [22], [43]–[45] to assess the probability of emergence for all resistance mutations identified in our screen (Fig. 6). The model shows that some substitutions, including L31I, F28T/C and Y93W, can cause failure of Daclatasvir monotherapy even with perfect treatment adherence. By incorporating parameter uncertainties into the analysis, we show that several other resistant mutants have substantial probabilities of arising (Fig. 6 and S5). If treatment is imperfect, many other escape mutants pose a risk of emergence, particularly if consecutive drug doses are missed. Some resistant mutations, such as Y93W, require two nucleotide changes from the WT sequence, and therefore are less likely to arise via natural adaptation from a single clone. Combination therapy is essential to prevent the growth and dissemination of resistant strains within or among infected individuals [46], and patients whose viral populations contain these mutations at high frequency would not benefit from Daclatasvir monotherapy. Thus, exploring the fitness and drug sensitivity of all possible single-mutant variants with saturation mutagenesis, combined with mathematical modeling, allows for risk assessment of possible evolutionary routes for the emergence of drug resistance. The approach shown here, combined with deep sequencing of clinical samples, would enable rational design of patient-specific combination therapies to minimize the threat of de novo resistance.

## Discussion

Modern molecular medicine has enormously accelerated the rate of developing novel therapeutics. For HCV, the establishment of *in vitro* replication systems has facilitated discovery of novel compounds that inhibit virus replication in addition to designed NS3 and NS5B inhibitors [18], [47]. This progress, however, creates a demand for more efficient methods to identify the inhibitory mechanisms of novel therapeutics as well as the determination of genetic barriers to resistance. The HCV-encoded non-enzymatic NS5A protein is a new target identified through chemical genetic screens [17]. Using the NS5A inhibitor Daclatasvir as an example, we have developed the novel qHRG method for systematically profiling drug-protein interactions. Unlike conventional virus adaptation studies where WT virus is passaged under drug treatment to positively select resistant mutations, we are capable of quantifying the drug sensitivity and fitness cost of all possible single amino acid mutations, thereby identifying the entire set of positions that govern particular drug-protein interactions. The profile of mutant fitness and drug sensitivity can be informative for the patient-specific use of Daclatasvir and may facilitate the development of second-generation drugs with higher genetic barriers.

NS5A is a multifunctional protein essential for several stages of HCV viral replication. It is a membrane-associated protein through an N-terminal amphipathic α-helix (amino acids 1–25), followed by an unstructured linker region (amino acids 26–35) and three functional domains. The N-terminal domain (domain I, amino acids 36–198) is the only structured domain [37], [48] and is targeted by a new class of direct-acting antivirals, such as Daclatasvir [20]. Although NS5A domain I appears to be a key regulator of viral replication [49] and is an effective drug target, its functional mechanisms are largely undefined.

The fitness landscape reveals novel strict requirements at positions critical for protein function. Projection of the fitness effects of mutations onto the structure illustrates the sequence-structure-function relationships, including the amino acid property requirements in the N-terminal amphipathic helix, the proline-rich unstructured linker region (residues 26–35), the conserved stretch of buried residues (87–91), and the putative RNA-binding residues essential for viral replication. Together, these results have demonstrated the specific residues critical for the multifunctional roles of NS5A in viral replication.

Since the discovery of the potent NS5A inhibitor, Daclatasvir, numerous long-term adaptation studies have been employed to identify drug resistant mutations and gain insight into the mechanisms of drug action [19], [20], [24], [26]. However, the positively selected drug-adaptive resistant clones identified previously only represent a fraction of the mutations that confer resistance. Furthermore, mutations that lead to hypersensitivity to Daclatasvir, which are equally informative, could not be identified in previous adaptation studies. Through qHRG analysis, we have quantitatively determined the drug sensitivity of all possible single amino acid variants in the domain IA of NS5A and thus provided a comprehensive understanding of the positions governing drug-protein interactions.

Structural analyses of the NS5A domain I previously revealed simultaneous existence of two different homodimer arrangements with non-overlapping interfaces on the opposite side of each monomer [37], [48]. Interestingly, in both forms of the dimer structure, the drug sensitivity-determining residues are located away from the dimerization interface, which supports previous results showing that the drug does not interfere with the dimerization of NS5A [23]. Instead, these residues cluster on the surface of domain 1A (Fig. 5B,C) and the unstructured linker region (amino acids 26–35) that connects the N-terminal amphipathic helix with the core of domain I. The existence of the two possible dimerization interfaces on the opposite side of the monomer has led to the prediction of superhelical array organization where the monomer is polymerized through alternative interfaces [48]. It is therefore possible that interaction with the drug may induce a conformation shift that disrupts the protein oligomerization, which prevents newly synthesized NS5A from facilitating replication complex formation.

Although the oligomerization hypothesis is supported by the extremely low working concentration of the drug even in replicon cell lines that homogenously harbor highly active replication complex, it does not exclude the possibility that the drug directly competes with cellular or viral factors for NS5A binding, and as a consequence, abolishes membranous web formation. In fact, a recent study suggests that the drug also affects genome replication in addition to virus assembly [22] possibly through inhibiting the function of NS5A that is required for genome replication. Several studies have shown that NS5A interacts with many host factors to hijack their cellular functions for facilitating viral replication. PI4KIIIα is a phosphatidylinositol 4-kinase identified as a host kinase targeted by NS5A and relocated to HCV replication complex [50]–[54]. Co-immunoprecipitation of NS5A deletion mutants and PI4KIIIα mapped the interaction to domain I [52], [53], and this interaction is critical for regulating the phosphorylation status of NS5A [55]. It is possible that the drug affects the interaction between NS5A and PI4KIIIα, and therefore obstructs genome replication. However, more direct evidence will be needed to elucidate the detailed mechanism.

**Figure 7:**
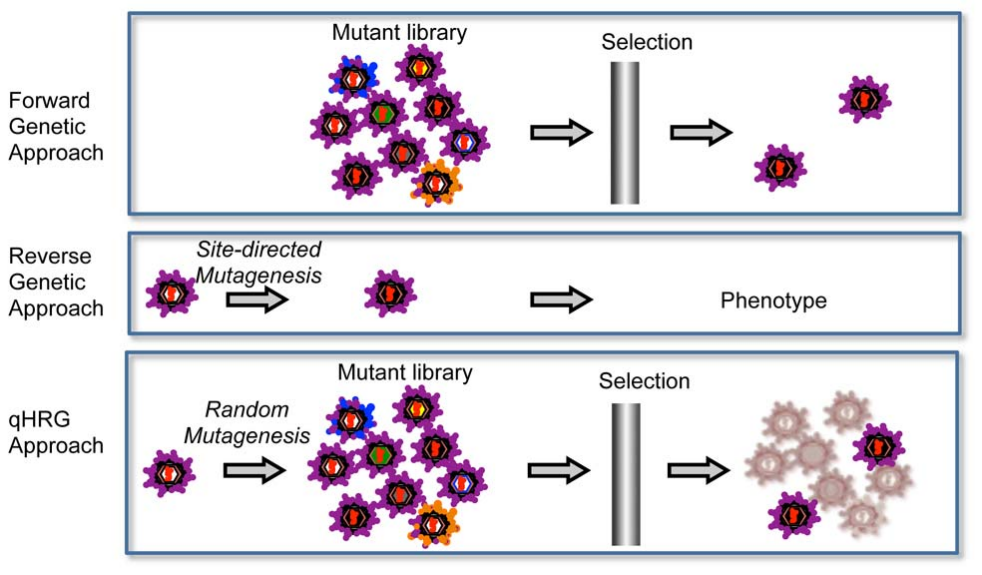
Comparison of qHRG platform with traditional genetics strategies. Forward genetics is usually done by selecting a random mutant library in a given condition for an aberrant phenotype. It is a powerful approach that is used to identify the responsible genes for the phenotype, but it is not suitable to characterize the negatively selected mutants. Reverse genetics is widely used to characterize the unknown function of a given gene and allows for illuminating both positively or negatively selected phenotypes, but it is typically limited to investigating a few mutations (*i.e.* it is low throughput). Our high-resolution genetic approach is a combination of forward and reverse genetics. The concept behind our approach is to randomly mutagenize the targeted gene at high resolution, potentially every base pair in a microbial genome, and by quantitatively analyzing the frequency of each variant before and after selection, we are able to determine the phenotypes of all variants in the population in parallel.

This systematic profiling approach is a combination of forward and reverse genetics, which we refer to as quantitative high-resolution genetics (qHRG) (Fig. 7). It fully utilizes recent advances in DNA sequencing capacity to accurately quantify the fitness of individual variants in a large and diverse population, thereby determine the phenotypic effects of each genetic modification within a single experimental platform. While this platform is limited to single mutant profiling, we found that the sum of individual mutational effects predicted multiple mutant sensitivity/resistance in 9 of 10 alternative genotypes reported by Scheel *et al.* [41]. Importantly, our ability to characterize mutations that led to increased drug sensitivity was essential in the prediction of multiple mutational effects. Furthermore, the simplicity and rapid time scale associated with this study enables investigation of mutational profiles in alternative mutant backgrounds. For example, if studying a protein-drug interaction system where all resistance mutations confer fitness costs, then qHRG could be used to conduct a secondary screen of a mutant library constructed on the background of the resistant mutations to investigate the existence of compensatory mutations and their likelihood of evolving.

Although our approach cannot identify context-dependent resistant mutations, *i.e.* those that are resistant only in the presence of an additional mutation, this study still identified most common mutations that contribute to escape in clinical specimens. Importantly, the major breakthrough amino acid substitutions conferring resistance in clinical trials were revealed by our qHRG approach (*e.g.* positions M28, Q30, L31 and Y93 for genotype 1a and L31 and Y93 for genotype 1b) [42], [56]. Interestingly, the vast majority of multiple-mutation variants in clinical specimens revealed by clonal sequencing analysis are combinations of these single amino acid substitutions [57]. Therefore, the systematic analysis of drug sensitivity/resistance of single mutations provides a reference for the viral response to Daclatasvir in HCV patients as analyzed by population sequencing. Moreover, likely due to the higher heterogeneity of natural HCV sequences *in vivo* compared to experimental replicons, the resistant mutation patterns in clinical specimens are more complex than those *in vitro* [57]. For example, some of the resistant mutations found in clinical samples were not previously detected *in vitro* (e.g. M28G, Q30G). However, the ability for these mutations to confer resistance was revealed by our complete drug profile (Fig. 4A).

This approach offers a quantitative view of the genetic barriers for all possible single amino acid changes during viral evolution, which can be linked to clinical outcome predictions using mathematical models. A profile of drug resistance may inform the rational use and combination of Daclatasvir based on viral mutant spectra from patients. With the continual reduction in cost and improvement in detection sensitivity of next-generation sequencing technology, direct sequencing of clinical specimens before treatment will reveal virus quasispecies in patients, ensuring timely diagnosis of pre-existing drug-resistant strains, enabling design of optimal therapeutic strategies for individuals. It will also enable monitoring of emergence of resistant strains during treatment to prevent the enrichment and spread of resistance. In addition, comprehensive mapping of genetic barriers to drug resistance will facilitate the development of second-generation drugs with higher fitness cost to resistance.

## Materials and Methods

### Cell culture, viruses and plasmids

The Huh-7.5.1 cell line was kindly provided by Dr. Francis Chisari from the Scripps Research Institute, La Jolla. The cells were cultured in Dulbecco's Modified Eagle Medium (DMEM, Invitrogen) supplemented with 10% of fetal bovine serum (FBS), 10 mM non-essential amino acids (Invitrogen, Carlsbad, USA), 10 mM HEPES, penicillin (100 units/ml), streptomycin (100 mg/ml), and 2 mM L-glutamine at 37°C with 5% CO_2_.

A plasmid of pFNX-HCV that carries the viral genome was synthesized in our lab based on the chimeric sequence of J6/JFH1 virus [13]. We introduced 7 nucleotide substitutions, resulting in synonymous mutations to the genome to distinguish this construct. The construct and sequence are available upon request.

### Construction of saturation mutant library in sub-domain IA of NS5A of HCV

The area to be mutated (86 amino acids) was divided into 5 small regions, with each composed of 17–18 amino acids. For each region, 17 (or 18) oligos, each of which contains one random codon (N_1_N_2_K, N_1_ and N_2_ code for A/T/G/C and K codes for T/G to ensure the coverage on every amino acid and minimize the possibility of getting stop codons) at the desired position were synthesized from IDT. This mutagenesis results in all possible amino acid substitutions at a given position to facilitate the functional exploration of each possible variant. The oligos each contain a BsaI recognition site on each end, which allows the generation of “sticky ends” to match the ends of each cassette. The cassettes were established by amplifying the fragments (from pFNX-HCV) flanking the desired mutation region with primers containing a BsaI recognition site, and digested with BsaI enzyme (NEB) to produce the “sticky ends” matching the oligos. The oligos and the cassettes were ligated with T4 DNA ligase (Invitrogen) overnight at 16°C and purified with PCR purification columns (Invitrogen). The ligated product was subcloned into the pFNX-HCV vector via BamHI and RsrII restriction sites and transformed. Approximately 50,000 colonies were collected for the library in total. Each library covers all possible mutations at approximately 50 fold.

### Selection of the saturation mutant library under drug treatment

The HCV NS5A inhibitor BMS-790052 was purchased from company Selleck Chemicals. The mutant virus library (12 ml) was used to infect naïve Huh-7.5.1 cells (4million) at M.O.I ∼0.2 with or without BMS-790052 treatment at 20 pM. The supernatant was collected at 144 hpi and used to infect naïve cells for the second round of selection. After two rounds of selection, the viral genome was recovered from the supernatant, and the mutated regions were PCR amplified and processed following the standard sample preparation protocol for HiSeq 2000 sequencing. Each library was tagged with a unique 6-bp molecular barcode sequence, which allows for the identification and study of relative fitness levels in each selection pool. The sequence of primers and barcodes can be found in the supplementary materials.

### Sequence mapping and data processing

Burrow-Wheeler Aligner was used to map the pair-end read by allowing 5 mismatches with a Q30 cutoff. Since both forward and reverse reads covered the whole randomized region, sequencing error was corrected by reads pairing. SAMtools and BamTools were employed for sequence analyses. Custom Python script was created for the other downstream data analyses.

### Determine the relative fitness score (*W*) of every mutant with regression analysis [28]

After determining the number of sequence reads (*Reads*) for each mutant, we then calculated the frequency of each mutant from each pool and the fitness score in relation to the WT. Any frequency that is lower than 0.0005 will be considered as noise and discarded, since the mutation frequency of HCV is about 10^−5^ to 10^−4^ nucleotide substitutions per nucleotide per round of genome replication [44]. The frequency of a given variant, *v*, in the pool #N (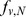) and the frequency of WT, *wt*, in the pool #N (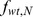) were calculated as follows:
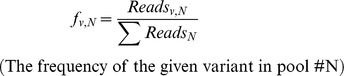


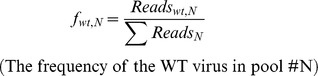
where 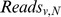 indicates the number of sequence reads for the variant (*v*) in pool #N, 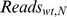 shows the number of sequence reads for the WT in pool #N, and 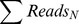 represents the total reads in the pool #N.

The relative fitness score of a given variant (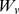) was determined as the antilogarithm of the slope of the regression:
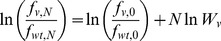
where 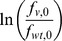
is the logarithm of the relative frequency of a given variant (*v*) in the input library (pool 0). The relative fitness score of each variant in drug treatment was calculated in the same way, but only with 2 rounds of selection in 20 pM drug treatment.

We then calculated the selection coefficient (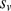):
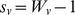
To examine the essentialness of each position, we also calculated the fold change of mutations at each position, which was used to color code the protein structure. The fraction at the i^th^ position (bearing j^th^ amino acid substitution, 19 total) in the pool N (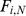) was
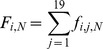
The fold change of mutations in pool #N compared to the input (pool 0) (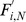) was:
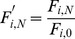



### Validation of mutant virus fitness

In total 16 individual mutant viruses containing point mutation as indicated in figure 2C were reconstructed based on a monocistronic Renilla luciferase HCV reporter virus, FNX24-RLuc, and recovered by electroporating the viral RNA genome into Huh-7.5.1 cells. The supernatant of transfected cells was collected and subjected to infect naïve Huh-7.5.1 cells for 2 passages. The replication of the viruses in each round was assayed by measuring the Renilla luciferase activity in the infected cells with Renilla Luciferase Assay System from Promega.

### Validation of the drug sensitivity of each mutant virus

10 individual mutant viruses, with a wide range of drug sensitivities in the screen, were reconstructed in order to determine their EC_50_. Each mutant, containing a point mutation as indicated in figure S4A, was constructed on the Renilla reporter virus background (FNX24-Rluc). The RNA was transfected into Huh7.5.1 cells to reconstitute the viruses, which were treated with different concentrations of Daclatasvir (0, 3 pM, 10 pM, 30 pM, 100 pM, 300 pM, 1000 pM, 3000 pM and 10 nM) at 96 hours post transfection. After 48-hour treatment of drug, the cells were collected and the replication of viruses in the cells was measured by Renilla Luciferase Assay System from Promega. The EC_50_ of each mutant was obtained by curve fitting using Prism 6 software.

### Mathematical modeling of the potential to develop drug resistance

To assess the potential to develop drug resistance for each mutant we considered in this study, we used mathematical models incorporating viral infection dynamics, pharmacodynamics of the drug, and level of drug adherence. We first assessed which mutants are resistant to the drug, *i.e.* that have potential to grow during long-term treatment. We then calculated the probabilities that those resistant mutants avoid extinction during the initial period of treatment (when target cells are depleted), such that they eventually lead to treatment failure. Please refer the supplementary information for detail.

## Supporting Information

Figure S1 **Functional profiling of NS5AD1A reveals the structure-function interactions of the domain.** (A) (B) The color-coded structure of domain NS5AD1A shows the essentialness of each residue. The fold change of mutations (log_10_) in pool 3 at each position was projected onto the structure with a blue-white-red color map in PyMOL. Blue color indicates a decreased frequency of mutations at a given position, and red color suggests an increased frequency of mutations. Grey color indicates the region that was not investigated in this study. (A) PDB structure 1ZH1 and (B) PDB structure 3FQM. The spectrum color bar is indicated below. (C) Hydrogen bonds of N82 with T87 and Q40 on PDB structure 3FQM.
(TIF)

Figure S2 **Residues 41 and 44 are critical for HCV genome replication and only form putative binding sites in one of the dimerization structures.** (A) Residues 41 and 44 locate at the basic groove of the NS5A dimer (PDB structure 1ZH1). The 4 residues are aligned perfectly in the dimer structure. Ribbon diagrams of three rotations of the domain I dimer (PDB structure 1ZH1) show residues 41 and 44 highlighted as green spheres. The basic groove was speculated to be an RNA-binding motif [37]. (B) The residues 41 and 44 are highlighted (PDB structure 3FQM).
(TIF)

Figure S3 **The fitness landscape of amino acids 18–103 in NS5A under drug treatment (20 mM).** (A) A heat map showing the profile of relative fitness under 20 mM of Daclatasvir treatment represented as selection coefficient under drug treatment (*s_*drug) for each variant *in vitro*. Color indicates the replication efficiency of each mutant under drug treatment calculated as *‘s*_drug*’* relative to WT. Red represents a positive *‘s’* (i.e. higher replication efficiency than WT under drug treatment) and blue stands for a negative *‘s’* (i.e. lower replication efficiency than WT under drug treatment). *s* = 0 means the same replication efficiency as the WT virus. The secondary structure of DIA is annotated below the figure (open circles: solvent exposed residues; filled circles: buried residues; half-filled circles: partially buried residues).
(TIF)

Figure S4 **Validation of the drug-sensitivity profiling results.** (A) Mutant viruses that varied in terms of resistance or sensitivity to Daclatasvir were reconstructed and their sensitivity to drug treatment was monitored individually under a series of concentrations of drug treatment. (B) The EC_50_ is calculated for each variant. (C) The EC_50_ values determined for individual variants are correlated strongly with the fold changes of fitness in the screen. The exponential relationship between EC_50_ and fitness fold change was utilized to approximate the EC_50_ of all mutants in the pool.
(TIF)

Figure S5 **Probability of mutants leading to therapeutic failure, and uncertainty analysis.** For each mutant, we integrated the uncertainties on parameter values by drawing parameter values at random from the distributions shown in Table S2. 1000 parameter sets were drawn for each of the 10 mutants identified as most likely to cause therapeutic failure. For a given parameter set, we calculated the probability that it would lead to therapeutic failure using Equation (S9), assuming perfect adherence to the recommended regimen of 60 mg Daclatasvir once daily. The distribution of the resulting probabilities of resistance is represented (in purple for mutants that are only one nucleotide mutation from the WT, green for the others). Different lateral scales were applied for the different mutations in order to show the relative patterns more clearly, but in all cases the set of bars should be normalized to represent the distribution of probabilities under the 1000 parameter sets. Strength of resistance and mutational distance interact to determine the probability that a given mutant will cause therapeutic failure. For instance, 93W has a higher EC_50_ than 28C, but because 93W is two mutations away from the WT it will be present at much lower frequency when treatment is initiated, and thus it is not the mutant with highest probability of causing therapeutic failure.
(TIF)

Table S1 **Summary of input mutant library properties.** (A) The size of each input library. The of bacterial colony counts gave an estimated coverage of each possible mutation (average number of times that each possible mutation was represented in the library). (B) Virus titers and MOIs used for each segment in the first round of infection. To maintain the coverage, we introduced 12 ml of infectious viruses with various titers, enough viruses that each variant in the plasmid library would be represented 10 times on average.
(TIF)

Table S2 **Description and values of parameters.** The pharmacokinetic parameters are set to reflect the current recommended drug regimen for Daclatasvir, 60 mg once daily [58], [59]. Note that only R_0_ and the pharmacokinetic parameters [43], [56], [60], [61] are required to compute the R_0_ contours in Fig. 6 in the main text.
(TIF)

Table S3 **Table of relative fitness scores of amino acids 18–103 in NS5A in virus replication that is used to plot** **figure 2A** **.**
(XLSX)

Table S4 **Table of relative fitness scores of amino acids 18–103 in NS5A under Daclatasvir treatment (20 pM) that is used to plot figure S3.**
(XLSX)

Table S5 **Table of fold change in relative fitness scores for each variant that is used to plot** **figure 4A** **.**
(XLSX)

Table S6 **Table of predicted EC_50_ values for each variant.**
(XLSX)

Text S1 **Supporting information for mathematical modeling of resistance development and treatment failure for Daclatasvir monotherapy.**
(DOCX)
