# Supplementary figures and images for: A Quantitative High-Resolution Genetic Profile Rapidly Identifies Sequence Determinants of Hepatitis C Viral Fitness and Drug Sensitivity
Source: PLoS Pathog. 2014 Apr 10;10(4):e1004064. doi: 10.1371/journal.ppat.1004064 (PMC3983061; doi:10.1371/journal.ppat.1004064)

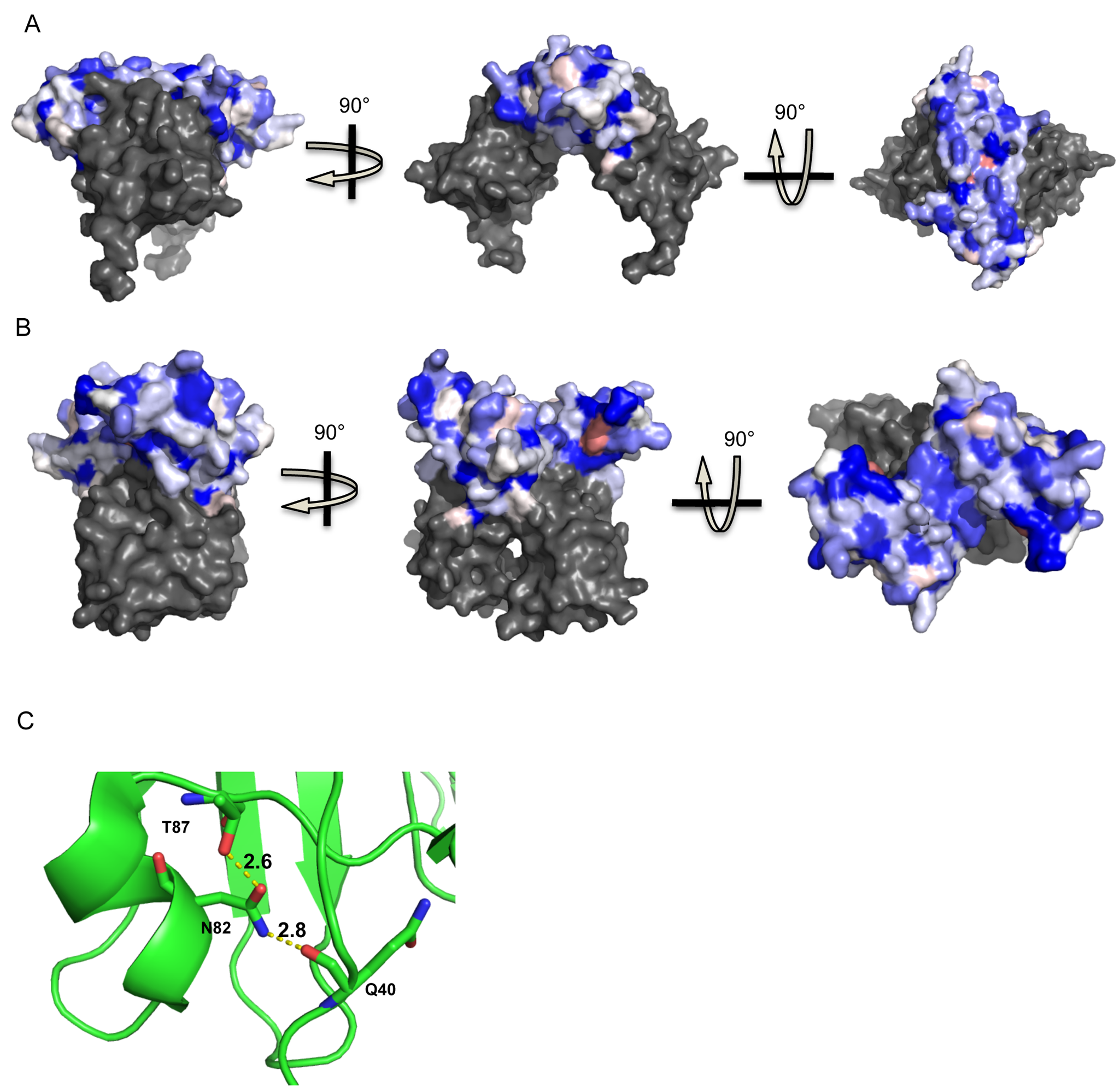

Supplement: Figure S1 — Functional profiling of NS5AD1A reveals the structure-function interactions of the domain. (A) (B) The color-coded structure of domain NS5AD1A shows the essentialness of each residue. The fold change of mutations (log10) in pool 3 at each position was projected onto the structure with a blue-white-red color map in PyMOL. Blue color indicates a decreased frequency of mutations at a given position, and red color suggests an increased frequency of mutations. Grey color indicates the region that was not investigated in this study. (A) PDB structure 1ZH1 and (B) PDB structure 3FQM. The spectrum color bar is indicated below. (C) Hydrogen bonds of N82 with T87 and Q40 on PDB structure 3FQM. (TIF) [file ppat.1004064.s001.tif]

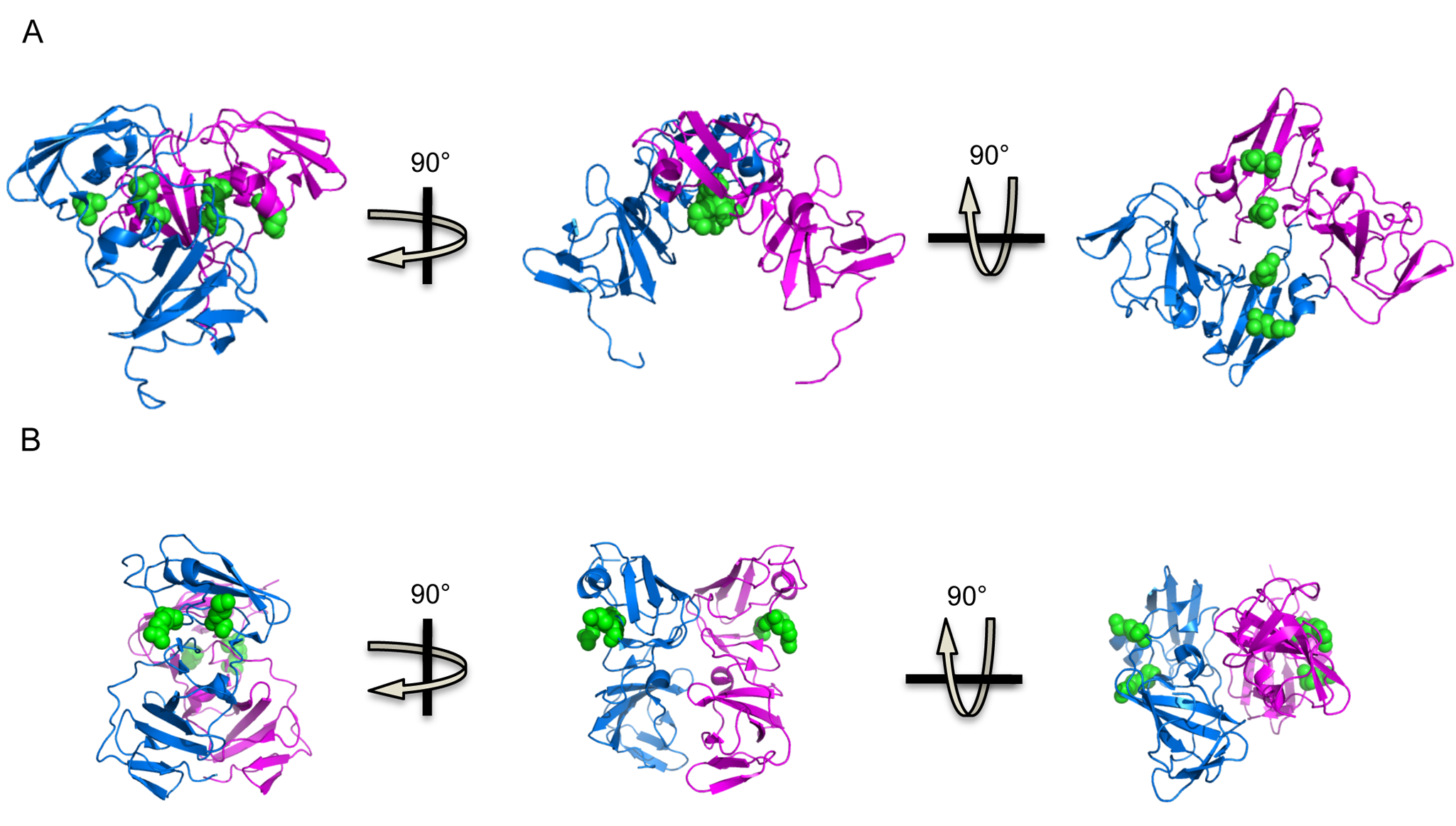

Supplement: Figure S2 — Residues 41 and 44 are critical for HCV genome replication and only form putative binding sites in one of the dimerization structures. (A) Residues 41 and 44 locate at the basic groove of the NS5A dimer (PDB structure 1ZH1). The 4 residues are aligned perfectly in the dimer structure. Ribbon diagrams of three rotations of the domain I dimer (PDB structure 1ZH1) show residues 41 and 44 highlighted as green spheres. The basic groove was speculated to be an RNA-binding motif [37]. (B) The residues 41 and 44 are highlighted (PDB structure 3FQM). (TIF) [file ppat.1004064.s002.tif]

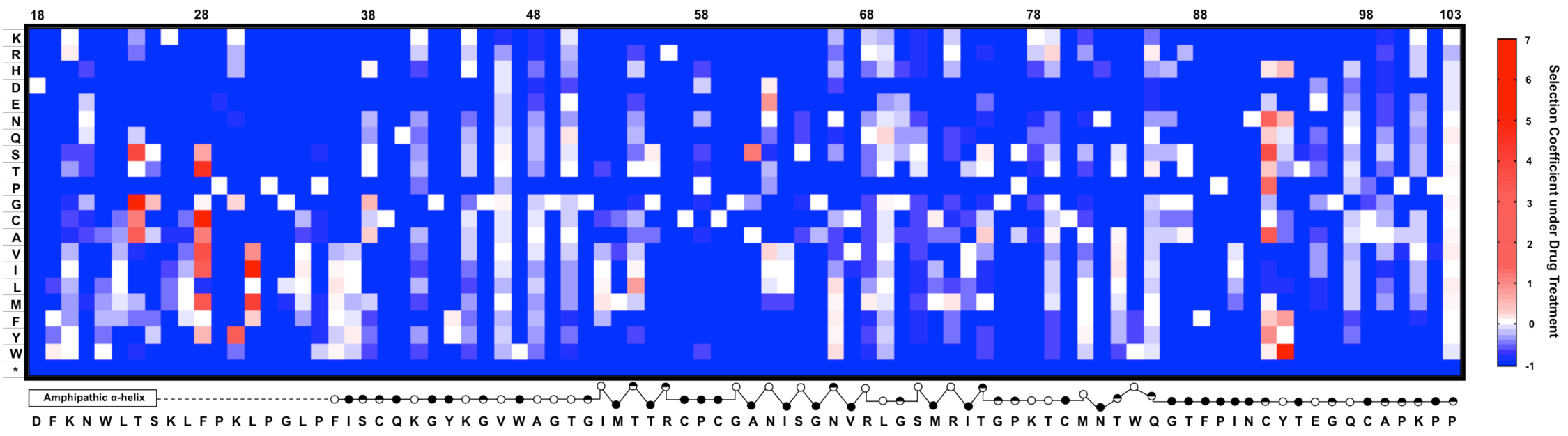

Supplement: Figure S3 — The fitness landscape of amino acids 18–103 in NS5A under drug treatment (20 mM). (A) A heat map showing the profile of relative fitness under 20 mM of Daclatasvir treatment represented as selection coefficient under drug treatment (s_drug) for each variant in vitro. Color indicates the replication efficiency of each mutant under drug treatment calculated as ‘s_drug’ relative to WT. Red represents a positive ‘s’ (i.e. higher replication efficiency than WT under drug treatment) and blue stands for a negative ‘s’ (i.e. lower replication efficiency than WT under drug treatment). s = 0 means the same replication efficiency as the WT virus. The secondary structure of DIA is annotated below the figure (open circles: solvent exposed residues; filled circles: buried residues; half-filled circles: partially buried residues). (TIF) [file ppat.1004064.s003.tif]

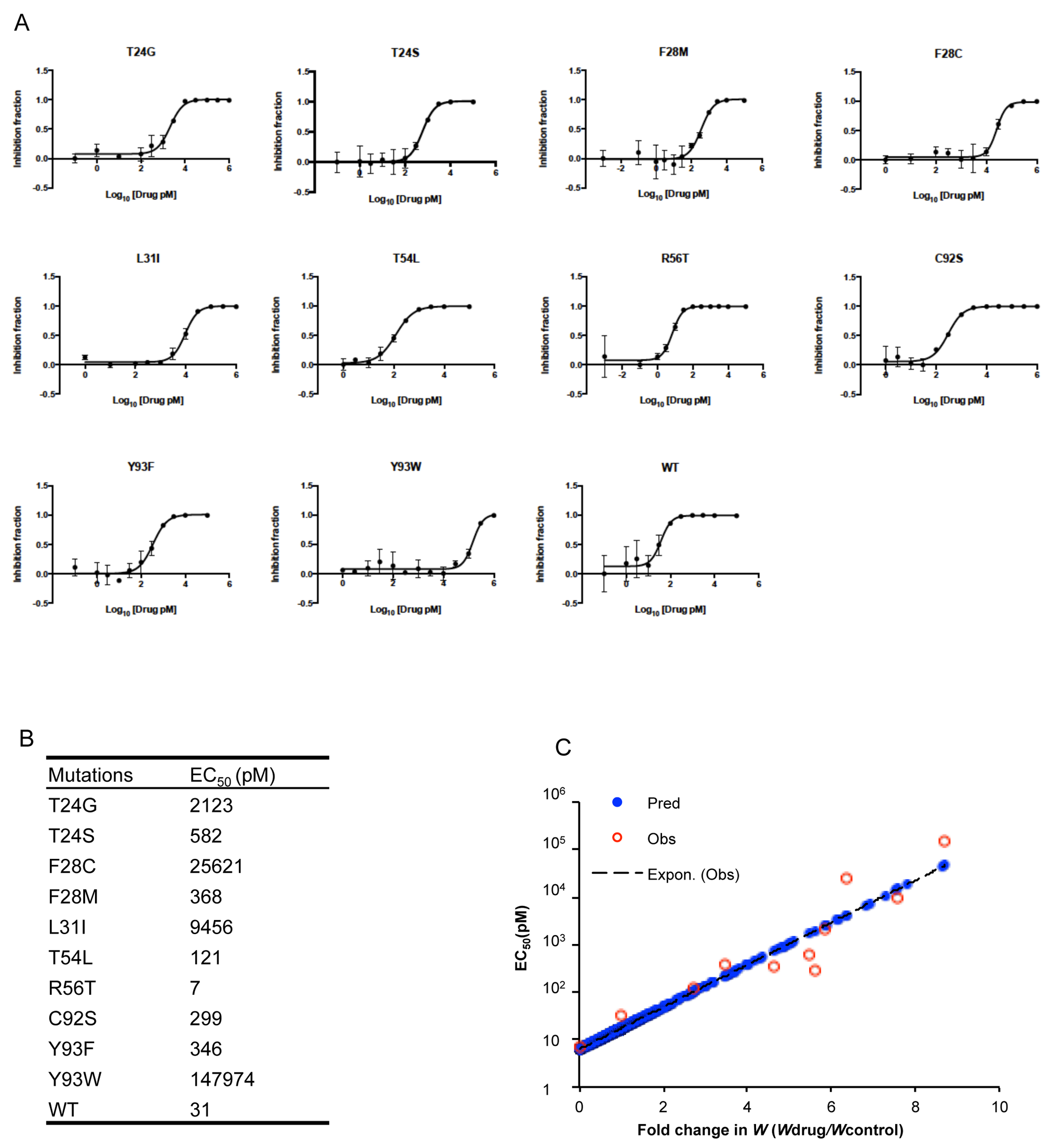

Supplement: Figure S4 — Validation of the drug-sensitivity profiling results. (A) Mutant viruses that varied in terms of resistance or sensitivity to Daclatasvir were reconstructed and their sensitivity to drug treatment was monitored individually under a series of concentrations of drug treatment. (B) The EC50 is calculated for each variant. (C) The EC50 values determined for individual variants are correlated strongly with the fold changes of fitness in the screen. The exponential relationship between EC50 and fitness fold change was utilized to approximate the EC50 of all mutants in the pool. (TIF) [file ppat.1004064.s004.tif]

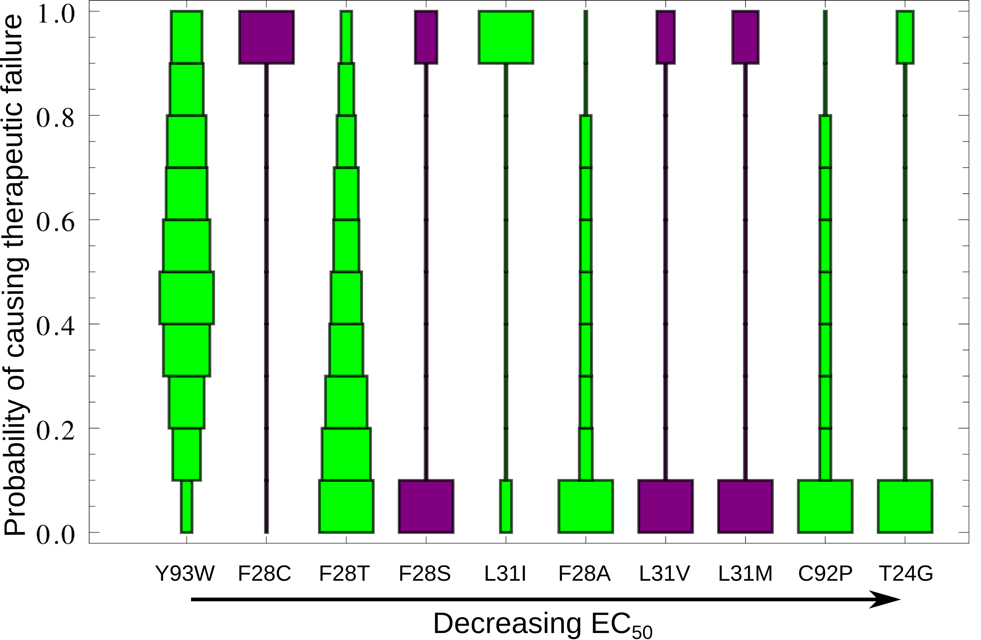

Supplement: Figure S5 — Probability of mutants leading to therapeutic failure, and uncertainty analysis. For each mutant, we integrated the uncertainties on parameter values by drawing parameter values at random from the distributions shown in Table S2. 1000 parameter sets were drawn for each of the 10 mutants identified as most likely to cause therapeutic failure. For a given parameter set, we calculated the probability that it would lead to therapeutic failure using Equation (S9), assuming perfect adherence to the recommended regimen of 60 mg Daclatasvir once daily. The distribution of the resulting probabilities of resistance is represented (in purple for mutants that are only one nucleotide mutation from the WT, green for the others). Different lateral scales were applied for the different mutations in order to show the relative patterns more clearly, but in all cases the set of bars should be normalized to represent the distribution of probabilities under the 1000 parameter sets. Strength of resistance and mutational distance interact to determine the probability that a given mutant will cause therapeutic failure. For instance, 93W has a higher EC50 than 28C, but because 93W is two mutations away from the WT it will be present at much lower frequency when treatment is initiated, and thus it is not the mutant with highest probability of causing therapeutic failure. (TIF) [file ppat.1004064.s005.tif]

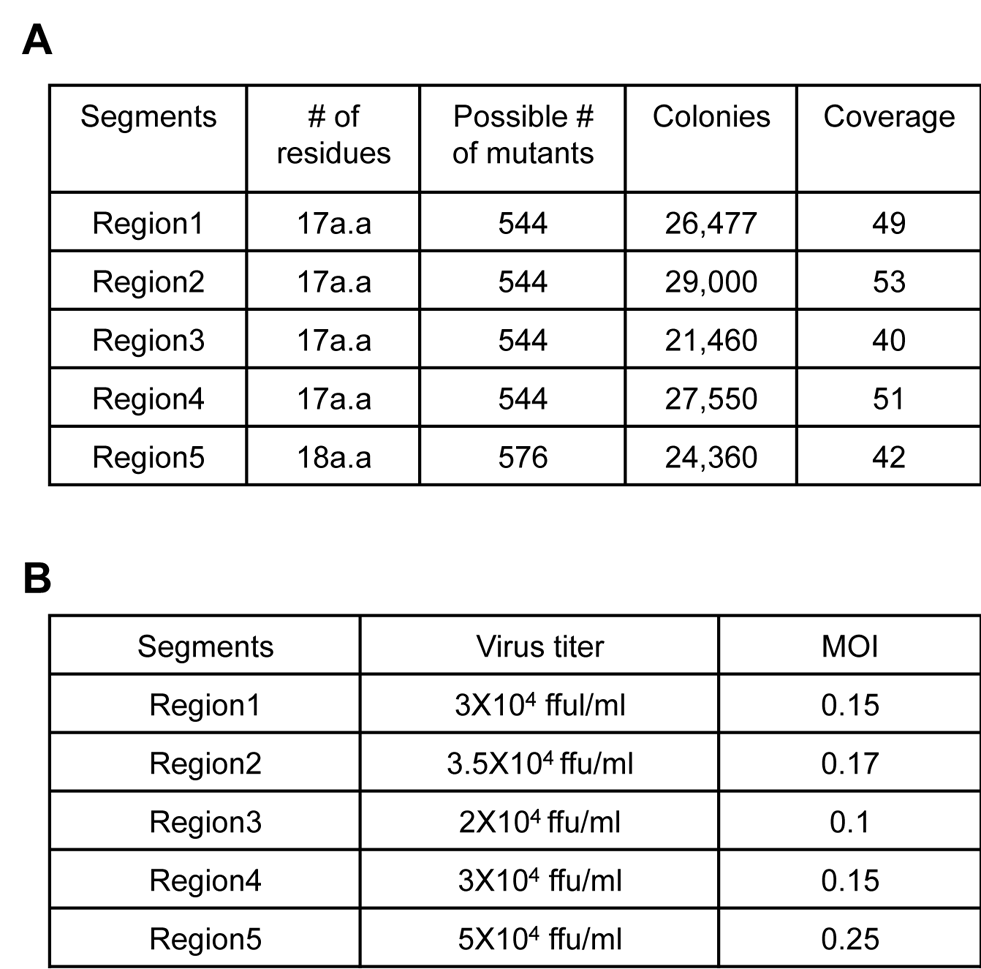

Supplement: Table S1 — Summary of input mutant library properties. (A) The size of each input library. The of bacterial colony counts gave an estimated coverage of each possible mutation (average number of times that each possible mutation was represented in the library). (B) Virus titers and MOIs used for each segment in the first round of infection. To maintain the coverage, we introduced 12 ml of infectious viruses with various titers, enough viruses that each variant in the plasmid library would be represented 10 times on average. (TIF) [file ppat.1004064.s006.tif]

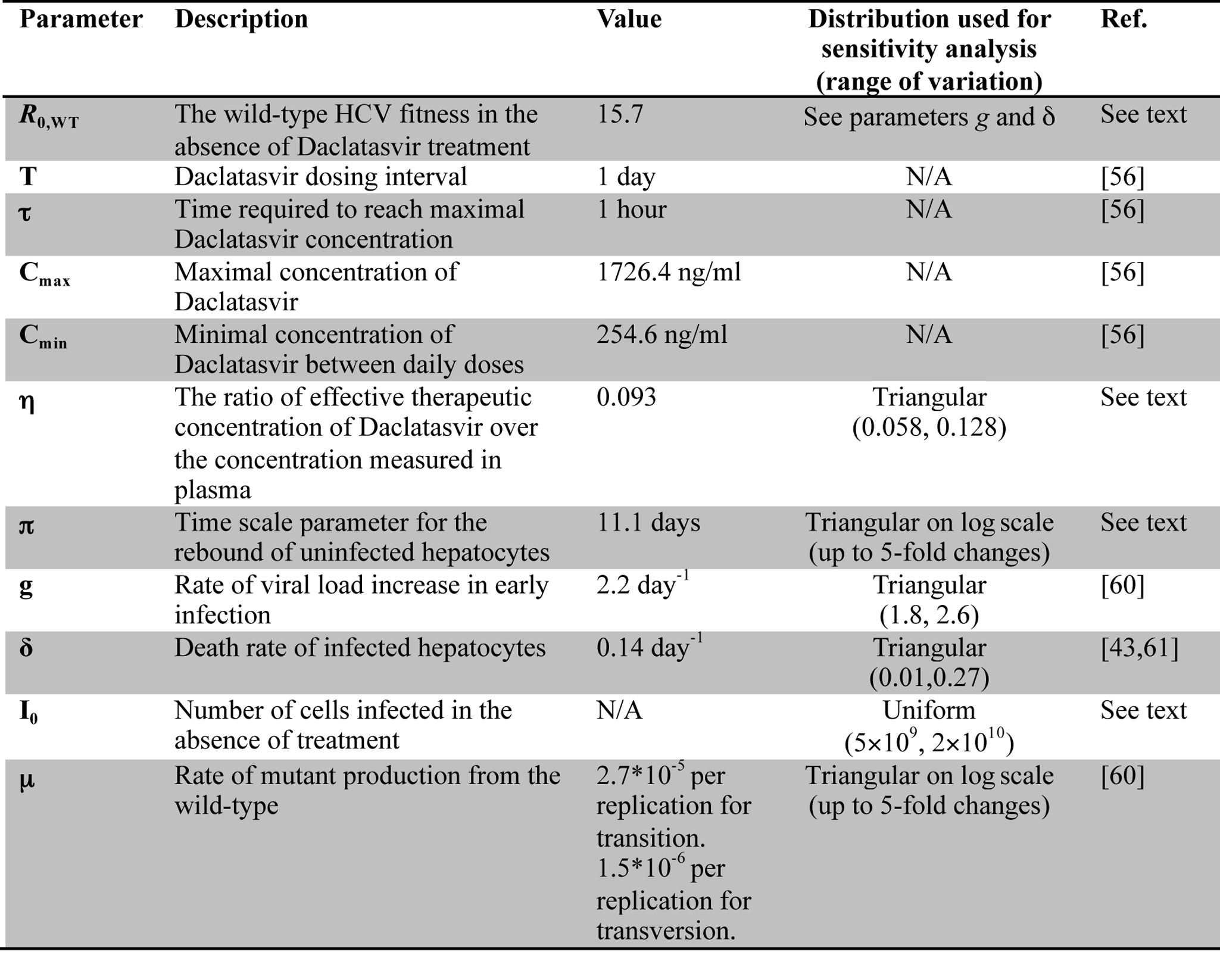

Supplement: Table S2 — Description and values of parameters. The pharmacokinetic parameters are set to reflect the current recommended drug regimen for Daclatasvir, 60 mg once daily [58], [59]. Note that only R0 and the pharmacokinetic parameters [43], [56], [60], [61] are required to compute the R0 contours in Fig. 6 in the main text. (TIF) [file ppat.1004064.s007.tif]
