# Supplementary material for: A Quantitative High-Resolution Genetic Profile Rapidly Identifies Sequence Determinants of Hepatitis C Viral Fitness and Drug Sensitivity
Source: PLoS Pathog. 2014 Apr 10;10(4):e1004064. doi: 10.1371/journal.ppat.1004064 (PMC3983061; doi:10.1371/journal.ppat.1004064)
Supplement: Text S1 — Supporting information for mathematical modeling of resistance development and treatment failure for Daclatasvir monotherapy. (DOCX) [file ppat.1004064.s012.docx]

Supporting Information

**Sequencing primer and barcodes used in HiSeq 2000.**

|  | PCR Primer Index | Barcode |
| --- | --- | --- |
| 1 | CAAGCAGAAGACGGCATACGAGAT**CGTGAT**GTGACTGGAGTTC | **ATCACG** |
| 2 | CAAGCAGAAGACGGCATACGAGAT**ACATCG**GTGACTGGAGTTC | **CGATGT** |
| 3 | CAAGCAGAAGACGGCATACGAGAT**GCCTAA**GTGACTGGAGTTC | **TTAGGC** |
| 4 | CAAGCAGAAGACGGCATACGAGAT**TGGTCA**GTGACTGGAGTTC | **TGACCA** |
| 5 | CAAGCAGAAGACGGCATACGAGAT**CACTGT**GTGACTGGAGTTC | **ACAGTG** |
| 6 | CAAGCAGAAGACGGCATACGAGAT**ATTGGC**GTGACTGGAGTTC | **GCCAAT** |
| 7 | CAAGCAGAAGACGGCATACGAGAT**GATCTG**GTGACTGGAGTTC | **CAGATC** |
| 8 | CAAGCAGAAGACGGCATACGAGAT**TCAAGT**GTGACTGGAGTTC | **ACTTGA** |
| 9 | CAAGCAGAAGACGGCATACGAGAT**CTGATC**GTGACTGGAGTTC | **GATCAG** |
| 10 | CAAGCAGAAGACGGCATACGAGAT**AAGCTA**GTGACTGGAGTTC | **TAGCTT** |
| 11 | CAAGCAGAAGACGGCATACGAGAT**GTAGCC**GTGACTGGAGTTC | **GGCTAC** |
| 12 | CAAGCAGAAGACGGCATACGAGAT**TACAAG**GTGACTGGAGTTC | **CTTGTA** |

**Mathematical modeling of the potential to develop drug resistance**

To assess the potential to develop drug resistance for each mutant we have considered in this study, we use mathematical models incorporating viral infection dynamics, pharmacodynamics of the drug, and level of drug adherence. We first assess which mutants are resistant to the drug, i.e. that have potential to grow during long-term treatment. We then calculated the probabilities that the resistant mutants avoid extinction during the initial period of treatment (when target cells are depleted), such that they eventually lead to treatment failure.

**a) Viral fitness**

A commonly used measure of viral fitness is the reproductive number, *R*_0_, which is the average number of infected cells produced by the first infected cell at the very beginning of an infection, and it is proportional to the rate of viral production in an infected cell. We define *R*_0,_*_WT_* to be the reproductive number of the wild-type virus in the absence of drug. In order to consider HCV mutants that may differ from the wild-type in their rate of viral production, we introduce a dimensionless parameter *W* to model the relative difference in fitness. Note that this parameter *W* corresponds to the fitness score measured in the experiments for each mutant.

Under drug treatment, the production of viruses from infected cells is reduced by *ε*(*t*), where *ε*(*t*) describes the level of viral inhibition due to drug treatment at time *t*. The value of *ε*(*t*) is a function of the drug concentration, *C*(*t*), the cooperativity of the drug, *h*, and the half maximal effective drug concentration of the HCV strain, *EC*_50_, and it is calculated as [1]:

(S1)

where our data suggest that *h*=1 for Daclatasvir (see the main text).

Thus the reproductive number of a mutant virus under drug treatment can be expressed as [1]:

$R_{0,drug}=(1-\varepsilon(t))\cdot W\cdot R_{0,WT}$. (S2)

#### Average drug inhibition (*ε_ave_*)

To calculate the mean viral fitness under drug treatment, we can use the average inhibition (*ε_ave_*) in place of *ε*(*t*), following a framework developed by Wahl and Nowak [1]. The average drug inhibition, *ε_ave_*, is dependent on the pharmacokinetics of the drug, the treatment adherence pattern of the patient, and the fitness of the mutant viral strain being considered.

First, we use mathematical equations to describe the drug concentration (*C*(*t*)) within a patient. The drug concentration typically follows a characteristic pattern: the drug concentration increases quickly to a peak level after taking a drug dose and then decreases exponentially until the next dose is taken [2]. Three pharmacokinetic parameters can be used to describe this pattern: the time to reach peak concentration after taking the drug (*τ*), the peak drug concentration (*C_max_*) and the minimum drug concentration before the next dose (*C_min_*). We use the following equation to describe the drug concentration between doses:

(S3)

where *T* is the interval between two consecutive doses, *w* is the decay rate of the drug, and .

Next, we adapt results from Wahl and Nowak[1] to calculate the average inhibition effect, *ε_ave_*, for different scenarios of drug dosing pattern in patients.

*Perfect adherence*

If the drug is taken perfectly according to the drug regimen, the inhibition effect of the drug is the same every dosing interval (after the initial period during which the drug concentration reaches equilibrium). Hence, the average inhibition effect of the drug over the course of drug therapy can be calculated by averaging over the inhibition effect of a single dosing interval (*T*):

(S4)

*Partial Adherence*

To model partial adherence, we consider the scenario in which doses are taken randomly as blocks of *n* doses with a fixed probability, *P*. For example, if *n*=1 and *P*=0.8, each dose is taken with an independent probability of 80%. Wahl and Nowak[1] showed that the average inhibition effect, *ε_ave_*(*n*,*P*), can be calculated as:

(S5)

where *A_j_* is the average inhibition effect during a dosing interval where *j* consecutive doses have been missed previously, and

(S6).

Note that the expression in Eqn. S5 is an infinite series. In the actual calculation, only the first 20 terms are calculated, because the later terms are negligibly small for the level of adherence investigated in this study, i.e. *P*= 0.6 and 0.8.

#### Defining regions of parameter space in which mutants are resistant (by calculating the *R*_0,_*_drug_*=1 contour)

From the theory above, it is possible to evaluate whether a mutant is able to grow in the long term under a given treatment regimen of Daclatasvir (i.e. whether *R*_0,_*_drug_*>1). Specifically, Eqn. S2 shows that *R*_0,_*_drug_* is a function of *W* and *ε_ave_*. In turn, *ε_ave_* is a function of the pharmacokinetics of the drug for a given level of adherence (*P*) and dosing pattern (*n*), and the strength of resistance of the mutant (represented by *EC*_50_; Eqns. S5 and S6).

To define the region of parameter space in which a mutant can grow under a given treatment regimen, we calculate the *R*_0,_*_drug_*=1 contour. By setting *R*_0,_*_drug_* to 1 in Eqn. S2, we can derive the minimum relative fitness, *W_min_*, required for a mutant to grow for a range of values of *EC*_50_:

(S7)

where *R*_0,_*_WT_* is the fitness of wild-type HCV in the absence of drug treatment and the expression for *ε_ave_*(*n*,*P*) is given in Eqns. S5 and S6.

Note that, to predict whether a mutant is resistant *in vivo* (i.e. to calculate the relevant value of *W*_min_), we need information about the effective therapeutic concentration of the drug at the site of action in the liver. We have estimated the effective therapeutic concentration based on viral load data observed in clinical trials[3], and found that the ratio of the effective therapeutic concentration to the drug concentration measured in plasma[2] is 0.093 (standard error: 0.035), i.e. the effective therapeutic concentration is roughly 10% of the concentration measured in plasma. Therefore, in the calculation of *ε_ave_* in Eqns. S4-S6, we employ a scaling parameter, *η*=0.093, to relate the drug concentrations measured in plasma to the effective therapeutic concentrations (*C*_max_ and *C*_min_): and , where *C*_max,_*_plasma_* and *C*_min,_*_plasma_* are the peak and trough drug concentration measured in plasma[4].

See Section e and Table S2 for the parameter values used to calculate the *R*_0,_*_drug_*=1 contours.

#### Probability of emergence of resistant mutants

Based on the analysis above, several mutants are potentially resistant (*R*_0,_*_drug_*>1) even if the drug is taken perfectly (Fig. 6 in main text). Because the number of target cells (hepatocytes) infected with HCV in an untreated patient is large, and the mutation rate of HCV is large, it is very likely that these mutants already exist in a patient before drug treatment is started[5]. If the rate at which cells get infected is proportional to the number of susceptible target cells, *H(t)*, then the effective reproductive number of the virus during treatment can be calculated as *R*_0,_*_drug_***H(t)*/*H*_0_ (with *H*_0_ the number of susceptible target cells in the absence of infection). Because many cells are infected at the initiation of treatment, there are few susceptible cells available, this effective reproductive number may initially be smaller than 1 even for a strongly resistant mutant. Thus the population of cells infected by the mutant virus may temporarily decrease before the target cells are replenished to a high enough level to allow it to grow. If the population of infected cells is small enough, the mutant lineage may go extinct. We now calculate the probability of survival of this mutant lineage, and thus ultimately the probability of therapeutic failure, adapting a model framework developed previously by Alexander and Bonhoeffer[6].

We first calculate the probability for extinction of a mutant lineage, *P_ext_*, using a hybrid model. The number of cells infected by the mutant virus is treated stochastically, with a fixed death rate *δ*. The rate of new infection can be expressed as *δ*R*_0_*_,drug_*H(t)/H*_0_ (see Ref. [6]). The number of uninfected hepatocytes increases rapidly upon drug treatment, due to the high efficacy of the drug against wild-type virus. The dynamics of target cells are calculated deterministically, assuming that they grow as if there were no new infections: infections by mutant viruses are negligible because they are in small numbers during this phase when they are at risk of extinction; infections by the wild-type viruses are negligible because the drug is very efficient at suppressing them. We further assume that target cells are produced at a rate *H*_0_/*π* and cleared at a per capita rate 1/*π*, and the ODE describing the dynamics of target cells is $\frac{dH}{dt}=\frac{1}{\pi}\cdot(H_{0}-H)$. Note that the parameter *π* here is a measure of the time scale of the rebound of target cells. Adapting the results of Alexander and Bonhoeffer[6], we find that *P_ext_* can be expressed in our model as:

(S8)

where $g_{0}=\frac{1}{\delta\cdot\pi}$, $g_{1}=g_{0}\cdot(R_{0,WT}-1)\cdot W\cdot(1-\varepsilon_{ave})$, $g_{2}=g_{0}\cdot(R_{0,WT}\cdot W\cdot(1-\varepsilon_{ave})-1)$, and is the generalized incomplete Gamma function.

If the mutant has greater fitness than the wild-type (*W*>1) in the absence of drugs, then most infected cells will carry the mutant and the total number of cells infected with the mutant before treatment is approximately the same as the total number of infected cells, *I*_0_. The probability of extinction of all mutant lineages is then $P_{ext}^{I_{0}}$. More often, the wild-type is fitter (*W*<1), and the resistant mutant is probably present but its abundance will be relatively low and subject to stochastic fluctuations. In this case the total number of infected cells, *I*_0_, will be comprised mostly of cells infected with the wild-type virus. If a mutant is produced at rate *μ* during replication of the wild type and (*1-W*)>>*μ*, the probability of survival of at least one mutant lineage is[6]:

$P_{all}=1-\left(\frac{1-W}{1-W\cdot P_{ext}}\right)^{\mu\cdot I_{0}/W}$ (S9)

Equation (S9) is used to calculate the distribution of probability of resistance for 1000 parameter sets randomly drawn from plausible biological ranges (shown in Table 2). The corresponding results are presented in Fig. S5

#### Estimation of parameter values

**Reproductive number (*R*_0,_*_WT_*), increase of the viral load in early infection (*g*) and death rate of infected cells (*δ*)**

The rate of increase of the viral load in early infection (*g*), which can be expressed as *R*_0,_*_WT_***δ*, has been estimated to be 2.2 ± 0.4 per day[7]. The death rate of infected cells (*δ*) is estimated to be 0.14 ± 0.13 per day[8,9]. The reproductive number is calculated directly from these quantities: *R*_0,_*_WT_* = g/*δ* = 2.2/0.14 = 15.7 in the Fig. 6 of the main text. For the uncertainty analysis in Fig. S4, we take *g* in a triangular distribution between 1.8 and 2.6 per day, and *δ* in a triangular distribution between 0.01 and 0.27 per day.

**Rate of mutant production from the wild-type (*μ*)**

The estimated mutation rate for HCV (*μ_tot_*) is about 3×10^-5^ per nucleotide per genome replication[7]. This rate is from one nucleotide to any of the other 3 nucleotides. One of these mutations is a transition, which is much more likely than the two transversions (estimated to be 18-fold more likely [7], thus the rates for the transition and each of the transversions are 0.9×*μ_tot_* and 0.05×*μ_tot_*, respectively). For each mutant, we add the contributions for all the mutational pathways from the wild-type to obtain the overall rate at which that mutant is produced from the wild-type. In the uncertainty analysis, we use *,* with *r* a random number drawn from a triangular distribution between -1 and 1, to explore changes up to 5-fold in this parameter.

**Number of infected cells before treatment (*I*_0_)**

We estimate the number of cells infected in the absence of treatment by two methods. On the one hand, 7% to 20% of hepatocytes in the liver of chronically infected patients are infected[10,11], and there are a total of roughly 10^11^ cells[7], leading to an estimated range of 7×10^9^ to 2×10^10^ infected cells. On the other hand, an infected cell produces about 200 virions per day [7], which are cleared at a rate of 22.7 per day[12]. The typical viral load is 10^6.5^ per mL in the plasma, with an extracellular total fluid (plasma and interstitial fluid) volume of about 15000mL [5]. Together, these elements result in an estimate of 5.5×10^9^ infected cells. Reassuringly, these estimates are of the same order of magnitude. In the uncertainty analysis, we use *I*_0_ drawn from a uniform distribution between 5×10^9^ and 2×10^10^.

**The time scale of rebound of uninfected hepatocytes (*π*)**

Rong *et al.*[5] have shown that the population of uninfected hepatocytes rebounds at a high rate after treatment begins, and they use an explicit term in their model to account for the rapid proliferation of uninfected hepatocytes. We use a simpler model (see Section d), in which the rate of hepatocyte proliferation is modeled using a time scale parameter *π*. We set the value of *π* such that the rebound rate of uninfected hepatocytes matches that reported in Rong *et al*.[5]. In the uncertainty analysis, we multiply this value by , with *r* drawn from a triangular distribution between -1 and 1.

**References:**

1. Wahl LM, Nowak MA (2000) Adherence and drug resistance: predictions for therapy outcome. Proceedings of the Royal Society of London Series B: Biological Sciences 267: 835-843.

2. Nettles RE, Gao M, Bifano M, Chung E, Persson A, et al. (2011) Multiple ascending dose study of BMS-790052, a nonstructural protein 5A replication complex inhibitor, in patients infected with hepatitis C virus genotype 1. Hepatology 54: 1956-1965.

3. Fridell RA, Wang C, Sun J-H, O'Boyle DR, Nower P, et al. (2011) Genotypic and phenotypic analysis of variants resistant to hepatitis C virus nonstructural protein 5A replication complex inhibitor BMS-790052 in Humans: In Vitro and In Vivo Correlations. Hepatology 54: 1924-1935.

4. Ke R, Loverdo C, Qi H, Olson CA, Wu NC, et al. (2013) Modelling clinical data shows active tissue concentration of daclatasvir is 10-fold lower than its plasma concentration. Journal of Antimicrobial Chemotherapy.

5. Rong L, Dahari H, Ribeiro RM, Perelson AS (2010) Rapid Emergence of Protease Inhibitor Resistance in Hepatitis C Virus. Science Translational Medicine 2: 30ra32.

6. Alexander HK, Bonhoeffer S (2012) Pre-existence and emergence of drug resistance in a generalized model of intra-host viral dynamics. Epidemics 4: 187-202.

7. Ribeiro RM, Li H, Wang S, Stoddard MB, Learn GH, et al. (2012) Quantifying the diversification of hepatitis C virus (HCV) during primary infection: estimates of the in vivo mutation rate. PLoS Pathog 8: e1002881.

8. Neumann AU, Lam NP, Dahari H, Gretch DR, Wiley TE, et al. (1998) Hepatitis C Viral Dynamics in Vivo and the Antiviral Efficacy of Interferon-Œ± Therapy. Science 282: 103-107.

9. Rong L, Guedj J, Dahari H, Coffield DJ, Jr., Levi M, et al. (2013) Analysis of hepatitis C virus decline during treatment with the protease inhibitor danoprevir using a multiscale model. PLoS Comput Biol 9: e1002959.

10. Liang Y, Shilagard T, Xiao SY, Snyder N, Lau D, et al. (2009) Visualizing hepatitis C virus infections in human liver by two-photon microscopy. Gastroenterology 137: 1448-1458.

11. Rodriguez-Inigo E, Lopez-Alcorocho JM, Bartolome J, Ortiz-Movilla N, Pardo M, et al. (2005) Percentage of hepatitis C virus-infected hepatocytes is a better predictor of response than serum viremia levels. J Mol Diagn 7: 535-543.

12. Guedj J, Dahari H, Rong L, Sansone ND, Nettles RE, et al. (2013) Modeling shows that the NS5A inhibitor daclatasvir has two modes of action and yields a shorter estimate of the hepatitis C virus half-life. Proceedings of the National Academy of Sciences 110: 3991-3996.

13. Tellinghuisen TL, Marcotrigiano J, Rice CM (2005) Structure of the zinc-binding domain of an essential component of the hepatitis C virus replicase. Nature 435: 374-379.

14. Nettles RE, Gao M, Bifano M, Chung E, Persson A, et al. (2011) Multiple ascending dose study of BMS-790052, a nonstructural protein 5A replication complex inhibitor, in patients infected with hepatitis C virus genotype 1. Hepatology 54: 1956-1965.

15. Neumann AU, Lam NP, Dahari H, Gretch DR, Wiley TE, et al. (1998) Hepatitis C viral dynamics in vivo and the antiviral efficacy of interferon-alpha therapy. Science 282: 103-107.

16. Pol S, Ghalib RH, Rustgi VK, Martorell C, Everson GT, et al. (2012) Daclatasvir for previously untreated chronic hepatitis C genotype-1 infection: a randomised, parallel-group, double-blind, placebo-controlled, dose-finding, phase 2a trial. Lancet Infect Dis 12: 671-677.

17. Lok AS, Gardiner DF, Lawitz E, Martorell C, Everson GT, et al. (2012) Preliminary study of two antiviral agents for hepatitis C genotype 1. N Engl J Med 366: 216-224.
